# Seven New Drimane-Type Sesquiterpenoids from Cultures of Fungus *Phellinus tuberculosus*

**DOI:** 10.1007/s13659-014-0002-x

**Published:** 2014-01-25

**Authors:** Jiang-Bo He, Tao Feng, Shen Zhang, Ze-Jun Dong, Zheng-Hui Li, Hua-Jie Zhu, Ji-Kai Liu

**Affiliations:** 1 State Key Laboratory of Phytochemistry and Plant Resources in West China, Kunming Institute of Botany, Chinese Academy of Sciences, Kunming, 650201 People’s Republic of China; 2 University of Chinese Academy of Sciences, Beijing, 100049 People’s Republic of China

**Keywords:** *Phellinus tuberculosus*, Drimane-type sesquiterpennoids, Phellinuins A–G

## Abstract

Seven new drimane-type sesquiterpennoids, phellinuins A–G (**1**–**7**), together with one known compound 3*β*,11,12-trihydroxydrimene (**8**) were isolated from the cultures of mushroom *Phellinus tuberculosus.* Their structures were elucidated on the basis of NMR and MS spectroscopic data and by comparison with data reported in the literature.

**Electronic supplementary material:**

The online version of this article (doi:10.1007/s13659-014-0002-x) contains supplementary material, which is available to authorized users.

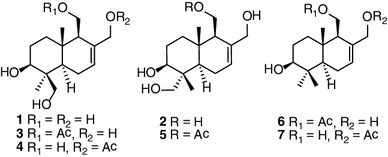

## Introduction

**Fig. 1:**
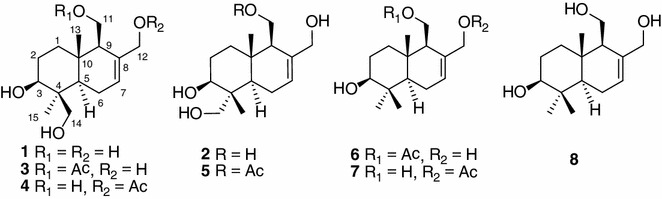
Structures of compounds **1**–**8**

*Phellinus* is a genus of fungi in the family Hymenochaetaceae. Many species cause white rot. Its fruiting bodies, often growing on wood, are resupinate, sessile, and perennial. The flesh is tough and woody or cork-like, and brown in color [1]. The fungus *Phellinus tuberculosus* has a wide distribution in Yunnan province of China [2]. The crude extract of mushroom *P. tuberculosus* was reported to possess antioxidant activity, which exhibited potent radical scavenging activity [3]. However, the chemical constituents of *P. tuberculosis* has not reported yet. As our continuous search for natural products from higher fungi [4–7], we carried out the chemical investigation on cultures of *P. tuberculosus*, which resulted in the isolation of seven new drimane-tpye sesquiterpennoids named phellinuins A–G (**1**–**7**) and one known compound (**8**) (Fig. 1). The structures of new compounds were determined on the basis of extensive spectroscopic analysis including NMR, MS, IR data, while the known compound was identified as 3*β*,11,12-trihydroxydrimene (**8**) by comparison with data reported in literature [8]. This paper describes their isolation and structural elucidation.

## Results and Discussion

**Fig. 2:**
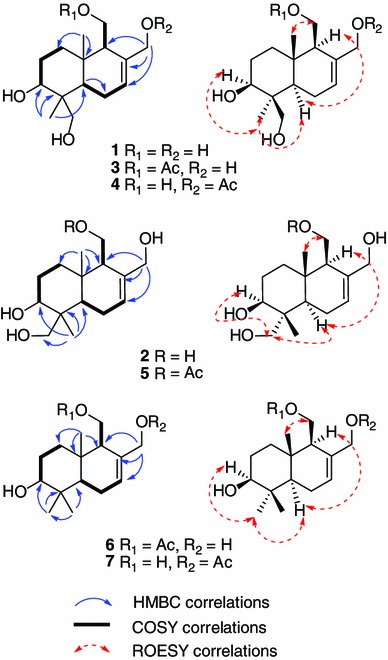
The main HMBC, COSY, and ROESY correlations of **1**–**7**

Compound **1** was obtained as a colorless oil. Its molecular formula C_15_H_26_O_4_ was revealed on the basis of the HREIMS at *m*/*z* 270.1827 (calcd for C_15_H_26_O_4_, 270.1831 [M]^+^), suggesting three degrees of unsaturation. The ^1^H NMR (Table 1) and ^13^C NMR data (Table 2) shows the presence of two methyls, six methylenes, four methines, and three quaternary carbons. In addition, the IR spectrum showed the presence of hydroxy group (3405 cm^−1^). Apart from one double bond, the remaining two degrees of unsaturation in **1** were assumed to be a bicyclic sesquiterpenoid. Detailed analysis of NMR data suggested that compound **1** should be a drimane-type sesquiterpenoid with a similar planar structure to that of 3*α*,11,15-trihydroxydrimene [9]. Analysis of 2D NMR data suggested that only Me-12 was oxygenated into an oxymethylene in **1**, which was suggested by the HMBC correlations from *δ*_H_ 4.23 (1H, d, *J* = 11.8 Hz, H-12a) and 3.95 (1H, d, *J* = 11.8 Hz, H-12b) to *δ*_C_ 138.7 (s, C-8). The HMBC data further supported that the other parts of the planar structure of **1** were the same to those of 3*α*,11,15-trihydroxydrimene (Fig. 2) [9]. In the ROESY spectrum (Fig. 2), the correlation of H-5/Me-15 suggested that C-14 was *β* oriented, while the correlation of Me-15/H-3, as well as the constant coupling of H-3 (dd, *J* = 11.8, 3.6 Hz), indicated OH-3 to be *β* oriented. On the basis of these data, the ROESY correlations of H-5/H-9 and Me-13/H-11 indicated that H-9 was *α* oriented, while Me-13 was *β* oriented. Therefore, compound **1** was established as 3*β*,11,12,14-tetrahydroxydrimene and named as phellinuin A.

**Table 1:** ^1^H NMR data (600 MHz) of compounds **1**–**7** in methanol-*d*_4_

No.	1^a^	2^a^	3^a^	4^a^	5^a^	6^b^	7^a^
1	2.08, dt (13.8, 3.0)	2.03, overlap	2.08, overlap	2.08, overlap	2.00, m	2.01,m	2.03, m
	1.32, overlap	1.29, td (13.8, 3.6)	1.32, overlap	1.32, overlap	1.27, m	1.29, m	1.29, m
2	1.80, m	1.66, m	1.80, m	1.80, m	1.67, m	1.63, m	1.63, m
	1.75, m		1.75, m	1.75, m			
3	3.39, dd (11.8, 3.6)	3.65, dd (11.6, 4.2)	3.39, dd (11.7, 3.6)	3.39, dd (12.0, 3.6)	3.63, dd (12.0, 4.8)	3.20, dd (13.8, 7.8)	3.20, dd (12.6, 4.8)
5	1.35, dd (12.6, 4.8)	1.63, m	1.35, dd (12.6, 4.8)	1.35, dd (12.6, 4.8)	1.63, overlap	1.25, m	1.23, dd (10.8, 4.8)
6	2.16, m	2.04, overlap	2.16, m	2.16, m	2.03, overlap	2.08, m	2.07, m
	1.95, t (3.6)		1.97, t (3.6)	1.95, t (3.6)			
7	5.77, t (3.0)	5.79, br. s	5.82, br. s	5.82, br. s	5.83, br. s	5.85, br. s	5.84, br. s
9	2.05, overlap	2.07, overlap	2.23, br. s	1.99, overlap	2.24, br. s	2.22, br. s	1.97, br. s
11	3.83, dd (10.8, 3.0)	3.85, dd (10.8, 3.0)	4.30, dd (12.0, 4.2)	3.82, dd (10.8, 4.8)	4.32, dd (11.4, 4.2)	4.31, dd (11.4, 4.2)	3.82, dd (10.8, 3.6)
	3.62, dd (11.4, 7.2)	3.62, dd (11.4, 7.2)	4.20, overlap	3.58, dd (10.8, 6.6)	4.21, dd (11.4, 6.0)	4.20, dd (11.4, 6.0)	3.58, dd (10.8, 6.6)
12	4.23, d (11.8)	4.25, d (12.6)	4.05, d (12.6)	4.70, d (12.6)	4.06, d (12.6)	4.06, d (12.6)	4.70, d (12.6)
	3.95, d (11.8)	3.97, d (12.6)	3.95, d (12.6)	4.53, d (12.6)	3.97, d (12.6)	3.97, d (12.6)	4,54, d (12.6)
13	0.79, s	0.84, s	0.84, s	0.80, s	0.90, s	0.86, s	0.82, s
14	4.19, d (11.4)	0.77, s	4.19, d (11.4)	4.19, d (11.4)	0.80, s	0.87, s	0.86, s
	3.48, d (11.4)		3.48, d (11.4)	3.48, d (11.4)			
15	1.20, s	3.48, d (10.8)	1.20, s	1.20, s	3.49, d (10.8)	0.98, s	0.98, s
		3.25, dd (10.8)			3.25, d (10.8)		
OAc			2.01, s	2.01, s	2.02, s	2.01, s	2.03, s

^a^Spectra were measured at 600 MHz

^b^Spectra were measured at 500 MHz

**Table 2:** ^13^C NMR data of compounds **1**–**7** in methanol-*d*_4_

No.	1^a^	2^a^	3^a^	4^a^	5^a^	6^b^	7^a^
1	38.8, CH_2_	38.6, CH_2_	38.9, CH_2_	38.8, CH_2_	38.7, CH_2_	38.9, CH_2_	39.0, CH_2_
2	28.8, CH_2_	27.8, CH_2_	28.8, CH_2_	28.7, CH_2_	27.7, CH_2_	28.6, CH_2_	28.8, CH_2_
3	81.4, CH	73.7, CH	81.3, CH	81.3, CH	73.5, CH	79.5, CH	79.7, CH
4	43.1, C	43.6, C	43.1, C	43.0, C	43.6, C	39.8, C	39.9, C
5	51.7, CH	43.0, CH	51.6, CH	51.1, CH	43.0, CH	50.7, CH	50.8, CH
6	24.4, CH_2_	24.0, CH_2_	24.4, CH_2_	24.5, CH_2_	24.0, CH_2_	24.2, CH_2_	24.5, CH_2_
7	126.2, CH	126.3, CH	126.7, CH	128.8, CH	126.7, CH	126.7, CH	129.1, CH
8	138.7, C	138.5, C	137.4, C	134.4, C	137.2, C	137.2, C	134.3, C
9	55.8, CH	55.7, CH	52.2, CH	55.7, CH	52.1, CH	52.1, CH	55.8, CH
10	36.5, C	36.4, C	36.8, C	36.6, C	36.7, C	36.9, C	36.8, C
11	61.3, CH_2_	61.4, CH_2_	63.6, CH_2_	60.9, CH_2_	63.6, CH_2_	63.5, CH_2_	60.9, CH_2_
12	66.8, CH_2_	67.0, CH_2_	65.8, CH_2_	68.6, CH_2_	65.9, CH_2_	65.7, CH_2_	68.7, CH_2_
13	16.1, CH_3_	15.7, CH_3_	16.1, CH_3_	16.0, CH_3_	15.8, CH_3_	15.1, CH_3_	15.0, CH_3_
14	65.0, CH_2_	12.6, CH_3_	65.0, CH_2_	65.0, CH_2_	12.6, CH_3_	15.9, CH_3_	16.1, CH_3_
15	23.3, CH_3_	66.6, CH_2_	23.3, CH_3_	23.3, CH_3_	66.5, CH_2_	28.0, CH_3_	28.2, CH_3_
OAc			21.2, CH_3_	21.1, CH_3_	21.2, CH_3_	21.1, CH_3_	21.1, CH_3_
OAc			173.0, C	173.0, C	173.0, C	172.9, C	173.0, C

^a^Spectra were measured at 150 MHz

^b^Spectra were measured at 125 MHz

Compound **2** was isolated as amorphous powder. The molecular formula was established to be C_15_H_26_O_4_ on the basis of the HREIMS at *m*/*z* 270.1828 (calcd for C_15_H_26_O_4_, 270.1831 [M]^+^). The 1D NMR data (Tables 1 and 2) were very similar to those of compound **1**, which indicated that both compounds had the same structure. However, the ROESY correlations of H-5/H-15 and H-15/H-3 indicated that Me-15 was oxygenated into an oxymethylene in **2**, while C-14 should be a methyl (Fig. 2). Detailed analyses of other 2D NMR data suggested that the other parts of **2** were the same to those of **1**. Therefore, the structure of compound **2** was established as 3*β*,11,12,15-tetrahydroxydrimene and named phellinuin B.

Compound **3**, a colorless oil, possessed a molecular formula C_17_H_28_O_5_, as deduced from HREIMS at *m*/*z* 312.1935 (calcd for C_17_H_28_O_5_, 312.1937 [M]^+^). The IR spectrum displayed the absorption bands for C=O (1722 cm^−1^), OH (3418 cm^−1^), and C=C (1642 cm^−1^). All the NMR data suggested that compound **3** was closely related to **1** except one more *O*-acetyl group in **3**. The *O*-acetyl group was substituted at C-11—as revealed by HMBC correlations from *δ*_H_ 4.30 (1H, dd, *J* = 12.0, 4.2 Hz, H-11a) and 4.20 (1H, overlap, H-11b) to *δ*_C_ 173.0 (s, OAc) and 52.2 (d, C-9). The other 2D NMR data suggested that the other parts of **3** were the same to those of **1** (Fig. 2). Thus, the structure of compound **3** was elucidated as phellinuin C as shown in Fig. 1.

Compound **4** was also obtained as a colorless oil. HREIMS gave one pseudomolecular ion at *m*/*z* 312.1947 (calcd for C_17_H_28_O_5_, 312.1937). The 1D NMR data (Tables 1 and 2) were very similar with those of compound **3**. However, the HMBC correlations from *δ*_H_ 4.70 (1H, d, *J* = 12.6 Hz, H-12a) and 4.53 (1H, d, *J* = 12.6 Hz, H-12b) to *δ*_C_ 134.4 (s, C-8) and 173.0 (s, OAc) suggested that the *O*-acetyl group was substituted at C-12 in **4** rather than at C-11 in **3**. The other parts of structure **4** were established to be the same with those of **3** by 2D NMR correlations (Fig. 2). Therefore, compound **4** was identified as phellinuin D.

Compound **5** was established as an *O*-acetyl derivative of **2**, which was supported by the HMBC correlations from *δ*_H_ 4.32 (1H, dd, *J* = 11.4 and 4.2 Hz, H-11a) and 4.21 (1H, dd, *J* = 11.4 and 6.0 Hz, H-11b) to *δ*_C_ 137.2 (s, C-8) and 173.0 (s, OAc). The other 2D NMR data suggested that the others parts of **5** were the same to those of **2**. Therefore, the structure of compound **5** was established and named as phellinuin E.

Compounds **6** and **7** were identified as *O*-acetyl analogues of the known compound 3*β*,11,12-trihydroxydrimene (**8**) [8]. The HMBC data suggested that the *O*-acetyl group was substituted at C-11 in **6** and C-12 in **7**, respectively (Fig. 2). Analyses of other 2D NMR data suggested that the other parts were the same to those of **8** (Fig. 2). Therefore, the structures of compounds **6** and **7** were established and named as phellinuin F (**6**) and phellinuin G (**7**), respectively.

## Experimental Section

### General Experimental Procedures

Optical rotations were measured on a Jasco-P-1020 polarimeter. IR spectra were obtained by using a Bruker Tensor 27 FT-IR spectrometer with KBr pellets. NMR spectra were acquired with instruments of Avance Ш 600 and Bruker DRX-500. HREIMS were measured on a waters autoSpec Primier P776 instruments. Silica gel (200–300 mesh, Qingdao Marine Chemical Inc., China) and Sephadex LH-20 (Amersham Biosciences, Sweden) were used for column chromatography (CC). Fractions were monitored by TLC and spots were visualized by heating silica gel plates immersed in vanillin-H_2_SO_4_ in EtOH, in combination with Agilent 1200 series HPLC system (Eclipse XDB-C18 column, 5 μm, 4.6 × 150 mm).

### Material and Cultural Conditions

Fruiting bodies of *Phellinus tuberculosus* were collected at Jingdong, Yunnan Province, China in 2003 and identified by Prof. Zhu-Liang Yang of Kunming Institute of Botany, CAS. The voucher specimen (NO.CGBWSHF00118) was deposited at herbarium of Kunming Institute of Botany. Culture medium: glucose (5 %), pork peptone (0.15 %), yeast (0.5 %), KH_2_PO_4_ (0.05 %), MgSO_4_ (0.05 %), The initial pH was adjusted to 6.0, the fermentation was first carried out on an Erlenmeyer flask for 6 days till the mycelium biomass reached to the maximum. Later it was transferred to a fermentation tank (20 L) at 24 °C and 250 rpm for 20 days, ventilation was set to 1.0 vvm (vvm: air volume/culture volume/min).

### Extraction and Isolation

The culture broth (20 L) was concentrated under vacuum, extracted three times with EtOAc. The organic layer was evaporated in vacuum to give a crude extract (3.1 g), which was separated by Sephadex LH-20 (MeOH) CC to afford fractions A–C. Fraction B (2.5 g) was separated by reversed-phased C18 column (MeOH-H_2_O, 30–100 %) to give sub-fractions B1 and B5. The sub-fraction B1 (110 mg) was further purified by silica gel (CHCl_3_-MeOH, 20/1) to yield **1** (5.8 mg), Fraction B2 was separated by Sephadex LH-20 (MeOH) to obtain B2-1 (50 mg), which was further isolated and purified by silica gel CC (CHCl_3_-MeOH, 20/1) to obtain **2** (31.6 mg). Fraction B3 was purified by Sephadex LH-20 (MeOH) and silica gel (CHCl_3_-MeOH, 30/1) to get **5** (2.2 mg) and **8** (8.6 mg). The sub-fraction B4 was purified by preparative HPLC (MeOH-H_2_O, 40 %, 10 mL/min) to obtain **3** (7.9 mg) and **4** (2.5 mg). Fraction B5 was separated by Sephadex LH-20 (MeOH) column chromatography, then further separated on silica gel (CHCl_3_-MeOH, 30/1) to give **6** (17.4 mg) and **7** (1.0 mg).

**Phellinuin A (1)**: colorless oils, [α]_D_^17.3^ − 10.3 (*c* 0.1 MeOH); IR (KBr) *v*_max_ 3405, 2963, 2930, 2871, 1665, 1443 cm^−1^; ^1^H NMR data (see Table 1); ^13^C NMR data (see Table 2); ESIMS (neg.) *m*/*z* 539 [2M − H]^−^; HREIMS *m*/*z* 270.1827 [M]^+^ (calcd for C_15_H_26_O_4_, 270.1831).

**Phellinuin B (2)**: amorphous powder, [α]_D_^20.6^ 0 (*c* 0.3 MeOH); IR (KBr) *v*_max_ 3345, 2930, 2878, 1666, 1442, 1057, 1983 cm^−1^; ^1^H NMR data (see Table 1); ^13^C NMR data (see Table 2); ESIMS (pos.) *m*/*z* 539 [2M − H]^−^; HREIMS *m*/*z* 270.1828 [M]^+^ (calcd for C_15_H_26_O_4_, 270.1831).

**Phellinuin C (3)**: colorless oil, [α]_D_^20.6^ − 4.2 (*c* 0.2 MeOH); IR (KBr) *v*_max_ 3418, 2966, 2933, 2859, 1722, 1642, 1454, 1385, 1256 cm^−1^; ^1^H NMR data (see Table 1); ^13^C NMR data (see Table 2); ESIMS (pos.) *m*/*z* 335 [M + Na]^+^; HREIMS *m*/*z* 312.1935 [M]^+^ (calcd for C_17_H_28_O_5_, 312.1937).

**Phellinuin D (4)**: colorless oil, [α]_D_^20.8^ − 5.4 (*c* 0.3 MeOH); IR (KBr) *v*_max_ 3421, 2961, 2932, 2858, 1736, 1631, 1443, 1384, 1254 cm^−1^; ^1^H NMR data (see Table 1); ^13^C NMR data (see Table 2); ESIMS (pos.) *m*/*z* 335 [M + Na]^+^; HREIMS *m*/*z* 312.1947 [M]^+^ (calcd for C_17_H_28_O_5_, 312.1937).

**Phellinuin E (5)**: colorless oil, [α]_D_^21.1^ + 5.6 (*c* 0.002 MeOH); IR (KBr) *v*_max_ 3424, 2932, 2891, 1736, 1634, 1456 cm^−1^; ^1^H NMR data (see Table 1); ^13^C NMR data (see Table 2); ESIMS (pos.) *m*/*z* 335 [M + Na]^+^; HREIMS *m*/*z* 312.1930 [M]^+^ (calcd for C_17_H_28_O_5_, 312.1937).

**Phellinuin F (6)**: colorless oil, [α]_D_^20.4^ − 2.8 (*c* 0.4 MeOH); IR (KBr) *v*_max_ 3441, 2965,2931, 2857, 1737, 1639, 1442 cm^−1^; ^1^H NMR data (see Table 1); ^13^C NMR data (see Table 2); ESIMS (pos.) *m*/*z* 319 [M + Na]^+^; HREIMS *m*/*z* 296.1986 [M]^+^ (calcd for C_17_H_28_O_4_, 296.1988).

**Phellinuin G (7)**: colorless oil, [α]_D_^20.4^ − 6.5 (*c* 0.1 MeOH); IR (KBr) *v*_max_ 3442, 2933, 2869, 1721, 1665, 1460, 1254 cm^−1^; ^1^H NMR data (see Table 1); ^13^C NMR data (see Table 2); ESIMS (pos.) *m*/*z* 4319 [M + Na]^+^; HREIMS *m*/*z* 296.1982 [M]^+^ (calcd for C_17_H_28_O_4_, 296.1988).

## Electronic supplementary material

Below is the link to the electronic supplementary material.

Supplementary material 1 (PDF 7717 kb)
