# Supplementary material for: Seven New Drimane-Type Sesquiterpenoids from Cultures of Fungus Phellinus tuberculosus
Source: Nat Prod Bioprospect. 2014 Jan 25;4(1):21–5. doi: 10.1007/s13659-014-0002-x (PMC3956979; doi:10.1007/s13659-014-0002-x)
Supplement: Supplementary file 1 — Supplementary material 1 (PDF 7717 kb) [file 13659_2014_2_MOESM1_ESM.pdf]

Seven new drimane-tpye sesquiterpenoids from cultures of fungus *Phellinus tuberculosus*

Jiang-Bo He[ab], Tao Feng[a], Shen Zhang[ab], Ze-Jun Dong[a], Zheng-Hui Li[a], Hua-Jie Zhu[a], Ji-Kai Liu[a*]

[a]*State Key Laboratory of Phytochemistry and Plant Resources in West China, Kunming Instituent of Botany, Chinese Academy of Sciences, Kunming 650201, People's Republic of China, and* [b] *University of Chinese Academy of Sciences, Beijing 100049, People's Republic of China*

*Corresponding author. Email: jkliu@mail.kib.ac.cn; Tel.: +86-871-65216327; Fax: +86-871-65212285; Postal address: 132# Lanhei Road, Heilongtan, Kunming 650201, Yunnan, China.

Supplementary data

Figure S1. ^{1}H NMR (600 MHz, methanol-d_4) spectrum of phellinuin A (**1**)……..........................S4

Figure S2. ^{13}C NMR and DEPT (150 MHz, methanol-d_4) spectrum of phellinuin A (**1**)…............S4

Figure S3. HSQC spectrum of phellinuin A (**1**)……………………………………………...S5

Figure S4. HMBC spectrum of phellinuin A (**1**)…………………………………….............S5

Figure S5. ^{1}H-^{1}H COSY spectrum of phellinuin A (**1**)……………………………...…….. ..S6

Figure S6. ROESY spectrum of phellinuin A (**1**)………………………………... ……………S6

Figure S7. HREIMS of phellinuin A (**1**)……………………………………………………S7

Figure S8. ^{1}H NMR (600 MHz, methanol-d_4) spectrum of phellinuin B (**2**)……..........……… S7

Figure S9. ^{13}C NMR and DEPT (150 MHz, methanol-d_4) spectrum of phellinuin B (**2**) ………. .S8

Figure S10. HSQC spectrum of phellinuin B (**2**)…………………………………………………S8

Figure S11. HMBC spectrum of phellinuin B (**2**)……………………………………. S9

Figure S12. ^{1}H-^{1}H COSY spectrum of phellinuin B (**2**)………………………………..............S9

Figure S13. ROESY spectrum of phellinuin B (**2**)…………………………….....................S10

Figure S14. HREIMS of phellinuin B (**2**)………………………………………….............S10

Figure S15. ^{1}H NMR (600 MHz, methanol-d_4) spectrum of phellinuin C (**3**)……......................S11

Figure S16. ^{13}C NMR and DEPT (150 MHz, methanol-d_4) spectrum of phellinuin C (**3**)….........S11

Figure S17. HSQC spectrum of phellinuin C (**3**)………………………………………….…...S12

Figure S18. HMBC spectrum of phellinuin C (**3**)……………………………………….........S12

Figure S19. ^{1}H-^{1}H COSY spectrum of phellinuin C (**3**)…………………………...……........S13

Figure S20. ROESY spectrum of phellinuin C (**3**)…………………………...……... ....S13

Figure S21. HREIMS of phellinuin C (**3**)……………………………………………………S14

Figure S22. ^{1}H NMR (600 MHz, methanol-d_4) spectrum of phellinuin D (**4**)……..........………S14

Figure S23. ^{13}C NMR and DEPT (150 MHz, methanol-d_4) spectrum of phellinuin D (**4**)….........S15

Figure S24. HSQC spectrum of phellinuin D (**4**)……………………………………………….S15

Figure S25. HMBC spectrum of phellinuin D (**4**)…………………………………….........S16

Figure S26. ^{1}H-^{1}H COSY spectrum of phellinuin D (**4**)…………………………………...….....S16

Figure S27. ROESY spectrum of phellinuin D (**4**)……………………………...….. ……..S17

Figure S28. HREIMS of phellinuin D (**4**)……………………………………….............S17

Figure S29. ^{1}H NMR (600 MHz, methanol-d_4) spectrum of phellinuin E (**5**)……....................S18

Figure S30. ^{13}C NMR and DEPT (150 MHz, methanol-d_4) spectrum of phellinuin E (**5**) ……..S18

Figure S31. HSQC spectrum of phellinuin E (**5**)………………………………………………..S19

Figure S32. HMBC spectrum of phellinuin E (**5**)……………………………………...…...S19

Figure S33. ^{1}H-^{1}H COSY spectrum of phellinuin E (**5**)…………………………...….…... …S20

Figure S34. ROESY spectrum of phellinuin E (**5**)…………………………...……... ………S20

Figure S35. HREIMS of phellinuin E (**5**)……………………………………………………S21

Figure S36. ^{1}H NMR (500 MHz, methanol-d_4) spectrum of phellinuin F (**6**)……............…….S21

Figure S37. ^{13}C NMR and DEPT (125 MHz, methanol-d_4) spectrum of phellinuin F (**6**)….. .. ..S22

Figure S38. HSQC spectrum of phellinuin F (**6**)…………………………………..................S22

Figure S39. HMBC spectrum of phellinuin F (**6**)……………………………………….... …S23

Figure S40. ^{1}H-^{1}H COSY spectrum of phellinuin F (**6**)……………………...……... ………S23

Figure S41. ROESY spectrum of phellinuin F (**6**)……………………...……... ……………S24

Figure S42. HREIMS of phellinuin F (**6**)…………………………………………...……..S24

Figure S43. ^{1}H NMR (600 MHz, methanol-d_4) spectrum of phellinuin G (**7**)……..............…..S25

Figure S44. ^{13}C NMR and DEPT (150 MHz, methanol-d_4) spectrum of phellinuin G (**7**)……...S25

Figure S45. HSQC spectrum of phellinuin G (**7**)………………………………………………..S26

Figure S46. HMBC spectrum of phellinuin G (**7**)……………………………………….. ......S26

Figure S47. ^{1}H-^{1}H COSY spectrum of phellinuin G (**7**)………………………………............S27

Figure S48. ROESY spectrum of phellinuin G (**7**)………………………………...…......…...S27

Figure S49. HREIMS of phellinuin G (**7**)…………………………………………………........S28

Figure S1. ^{1}H NMR (600 MHz, methanol-d_4) spectrum of phellinuin A (**1**).

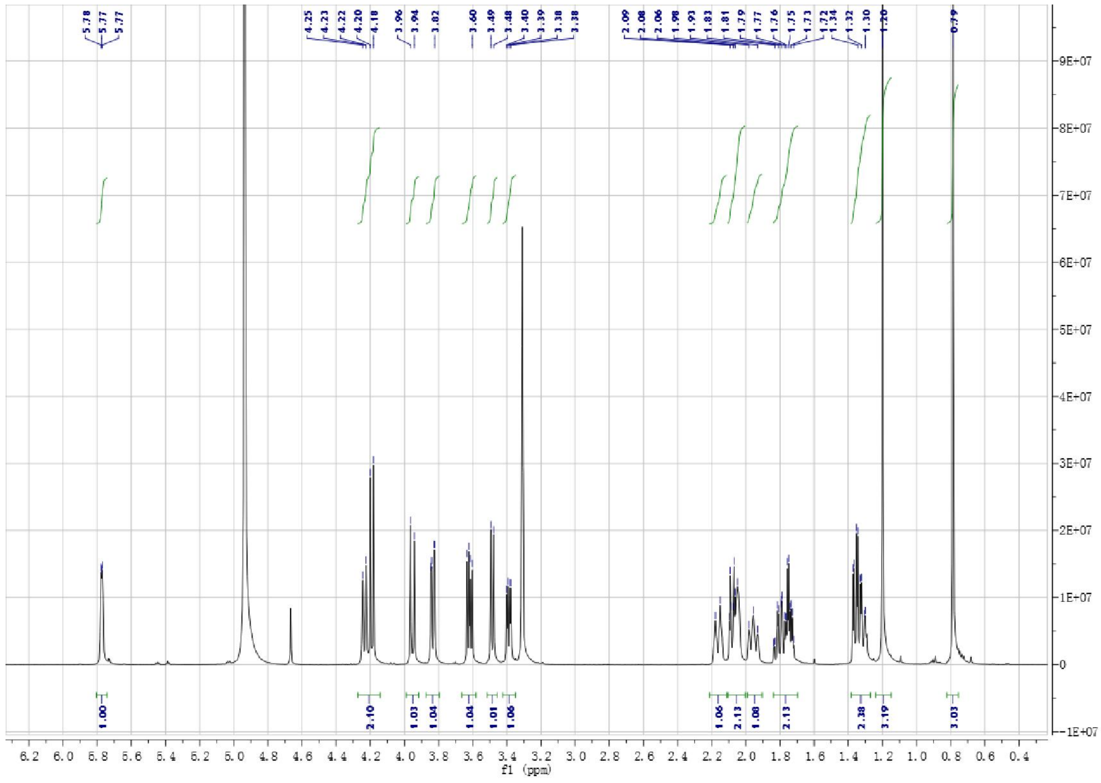

Figure S2. ^{13}C NMR and DEPT (150 MHz, methanol-d_4) spectrum of phellinuin A (**1**).

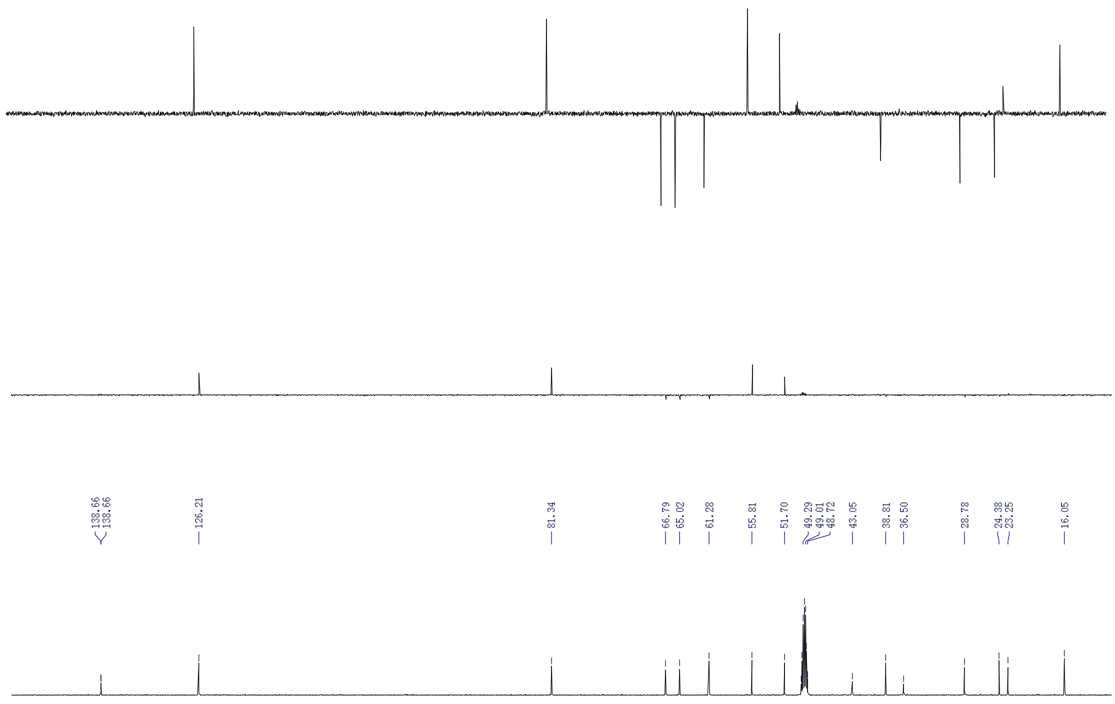

Figure S3. HSQC spectrum of phellinuin A (**1**).

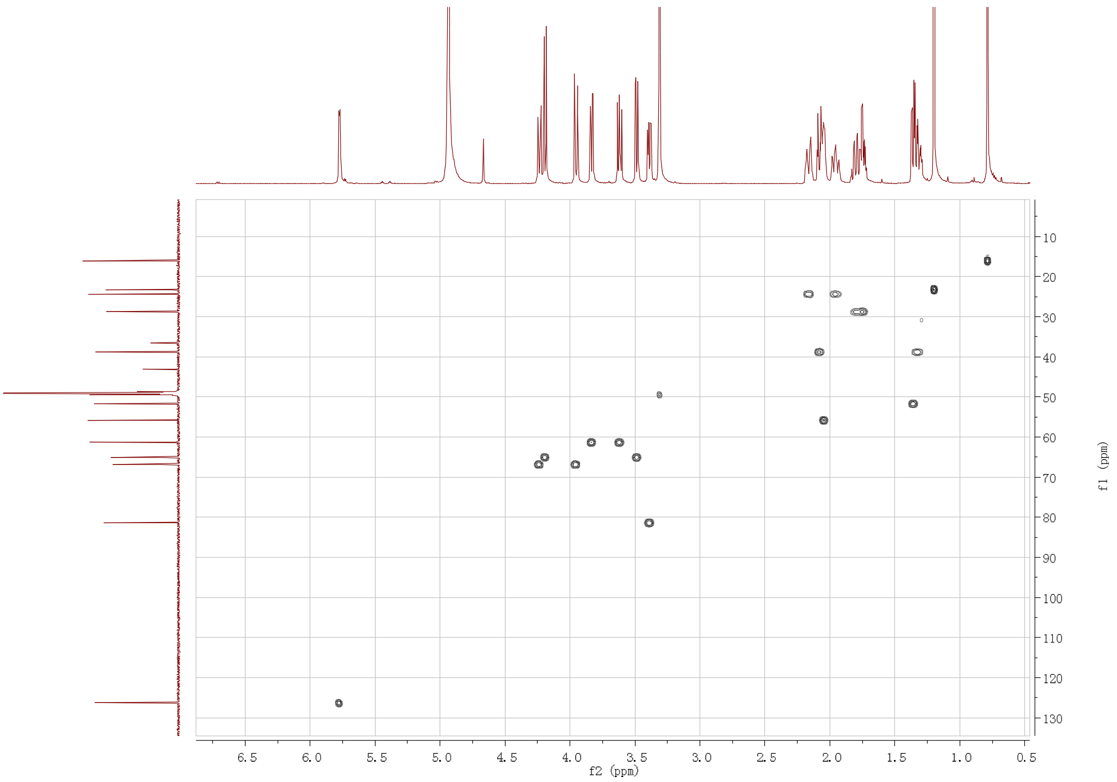

Figure S4. HMBC spectrum of phellinuin A (**1**).

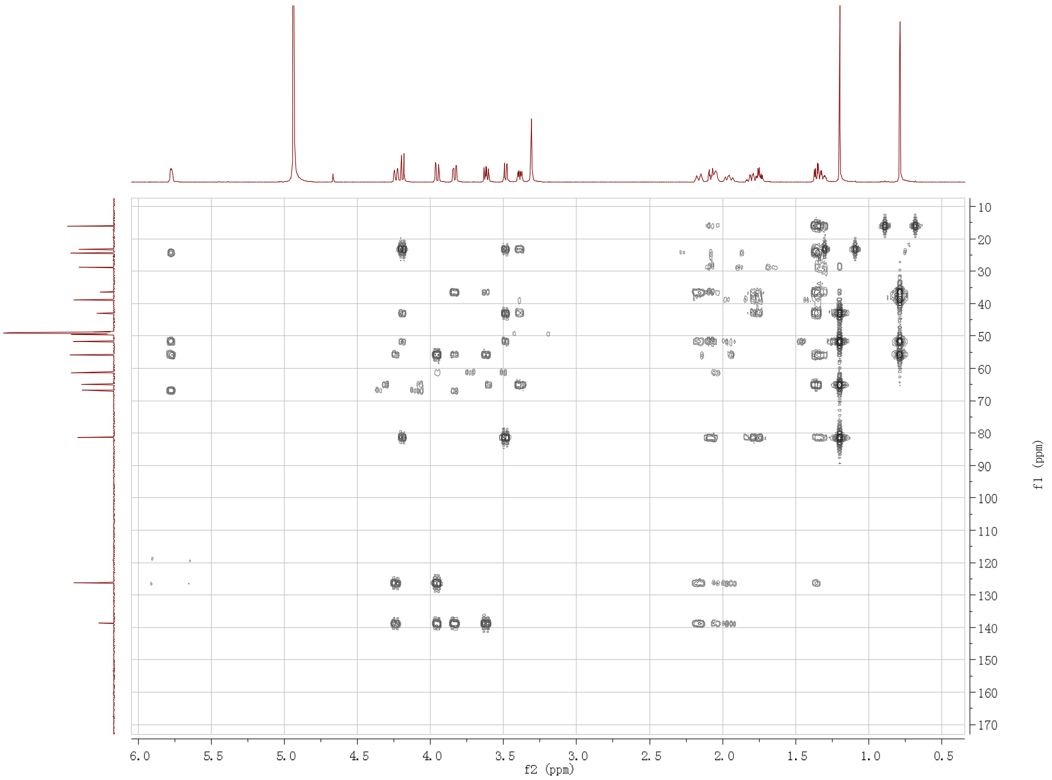

Figure S5. ^{1}H-^{1}H COSY spectrum of phellinuin A (**1**).

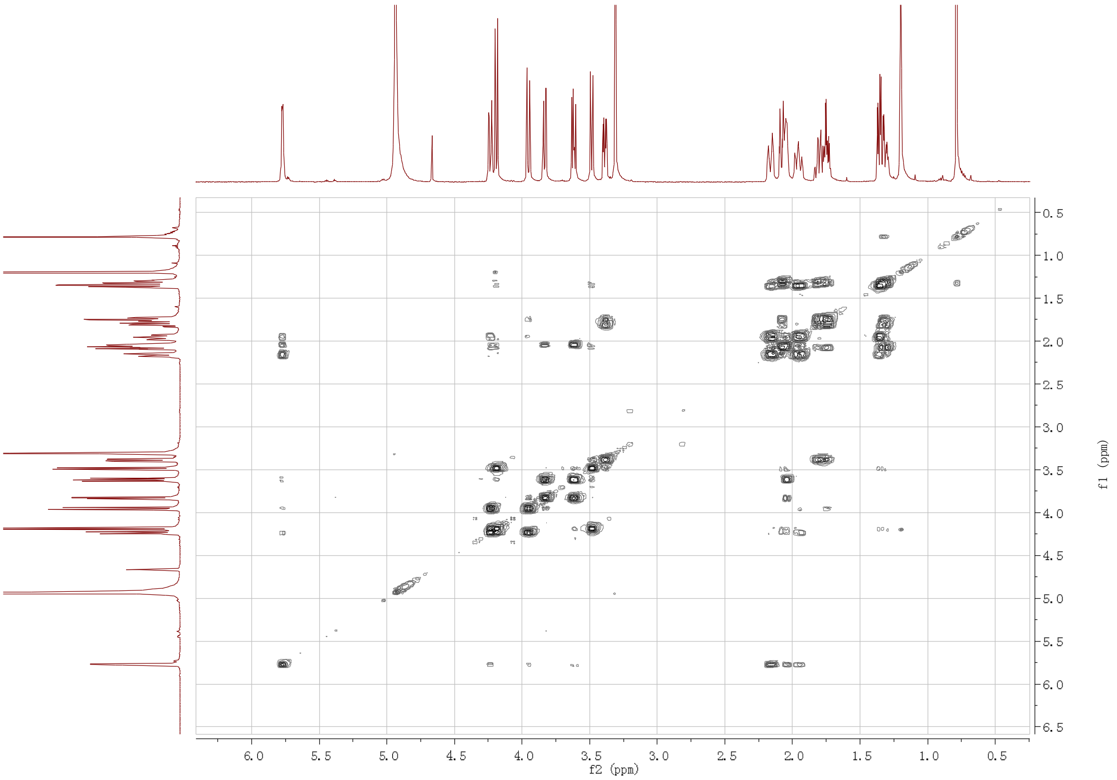

Figure S6. ROESY spectrum of phellinuin A (**1**).

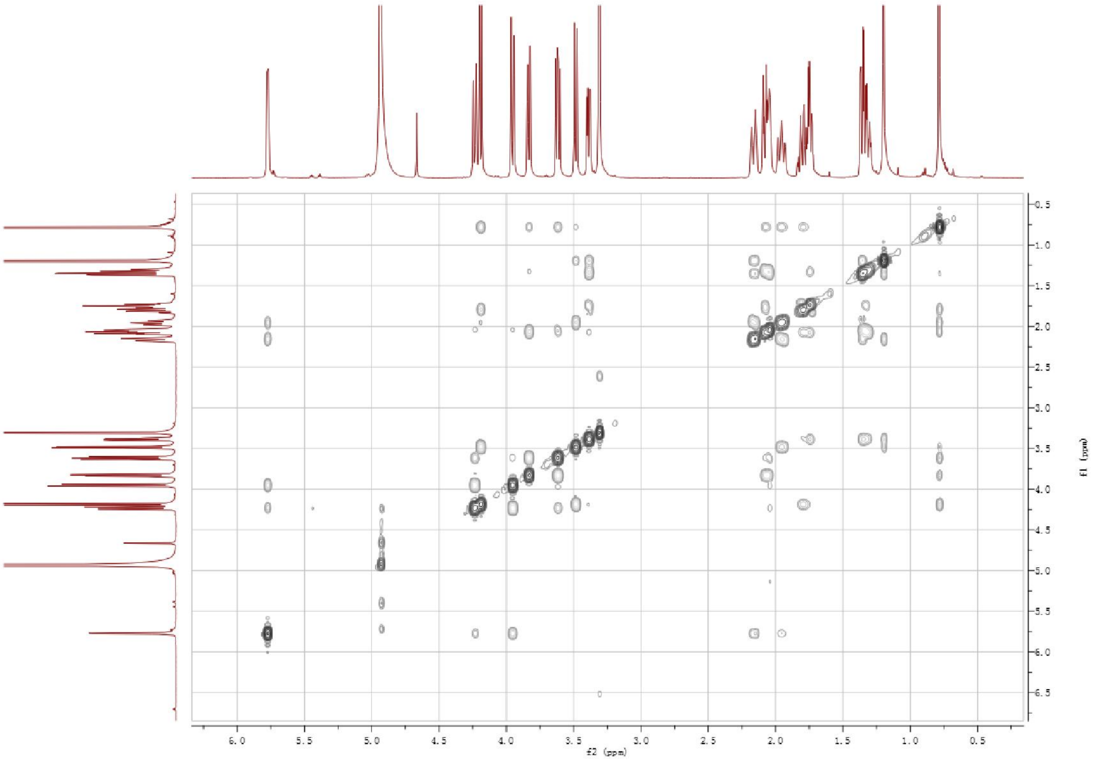

Figure S7. HREIMS of phellinuin A (**1**).

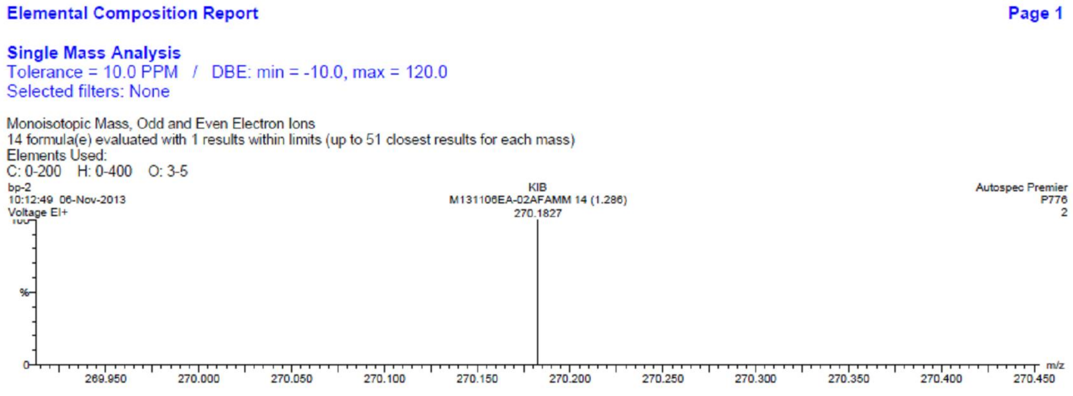

Minimum:				-10.0		
Maximum:		200.0	10.0	120.0		
Mass	Calc. Mass	mDa	PPM	DBE	i-FIT	Formula
270.1827	270.1831	-0.4	-1.5	3.0	5546025.5	C15 H26 O4

Figure S8. ^{1}H NMR (600 MHz, methanol-d_4) spectrum of phellinuin B (**2**).

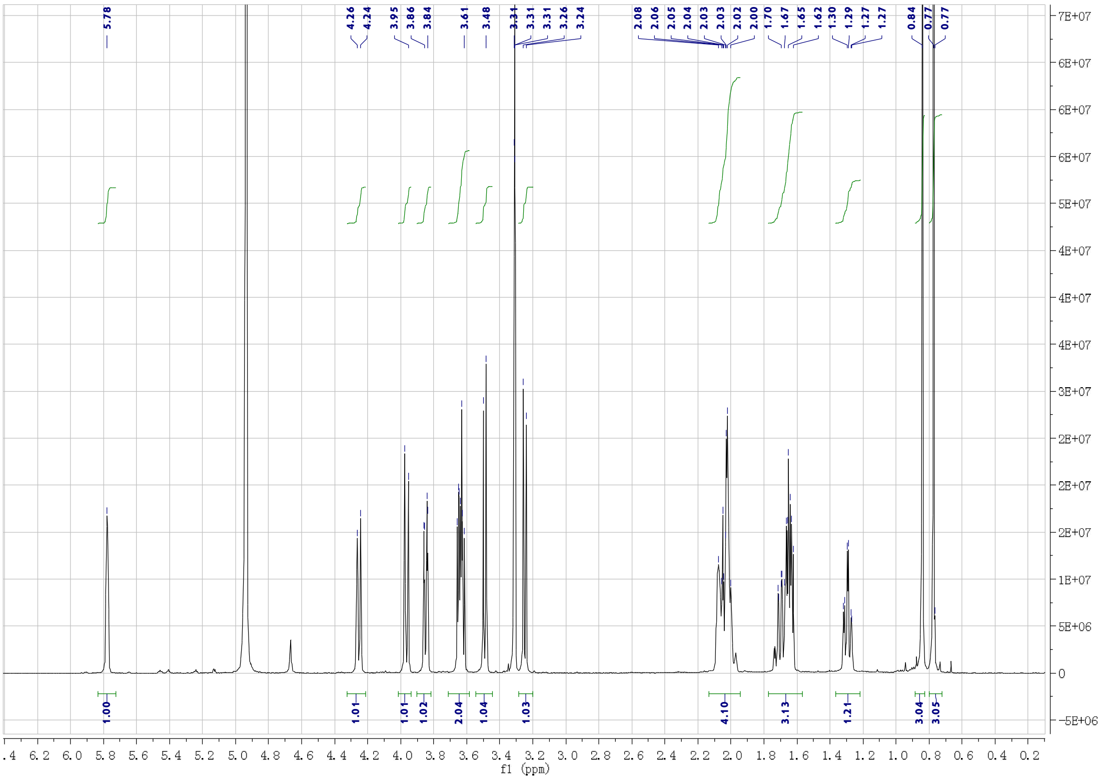

Figure S9. ^{13}C NMR and DEPT (150 MHz, methanol-d_4) spectrum of phellinuin B (**2**).

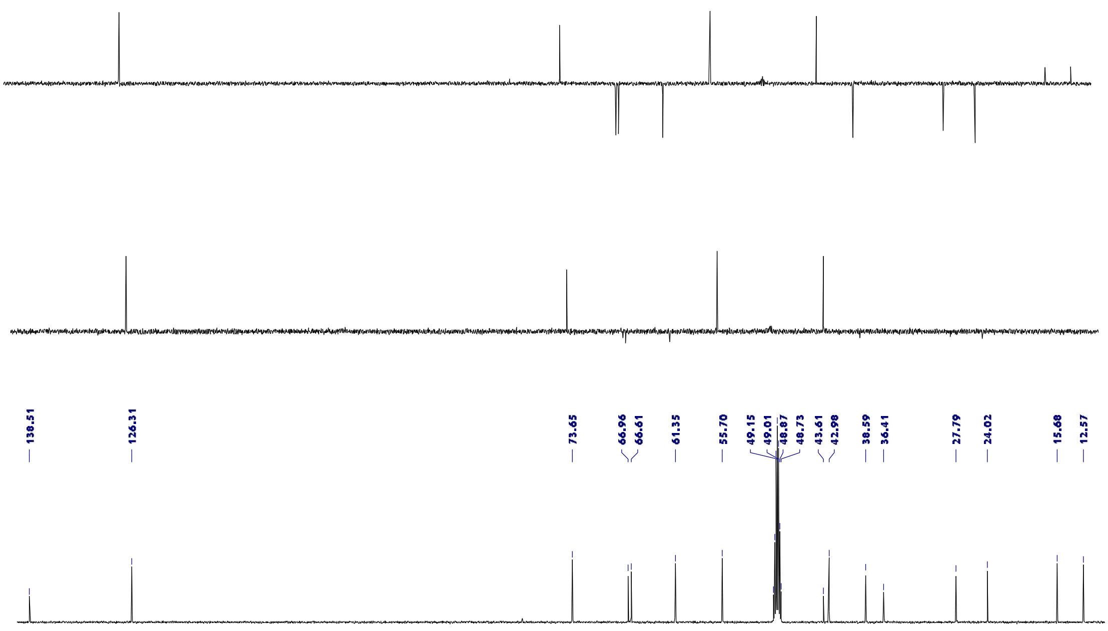

Figure S10. HSQC spectrum of phellinuin B (**2**).

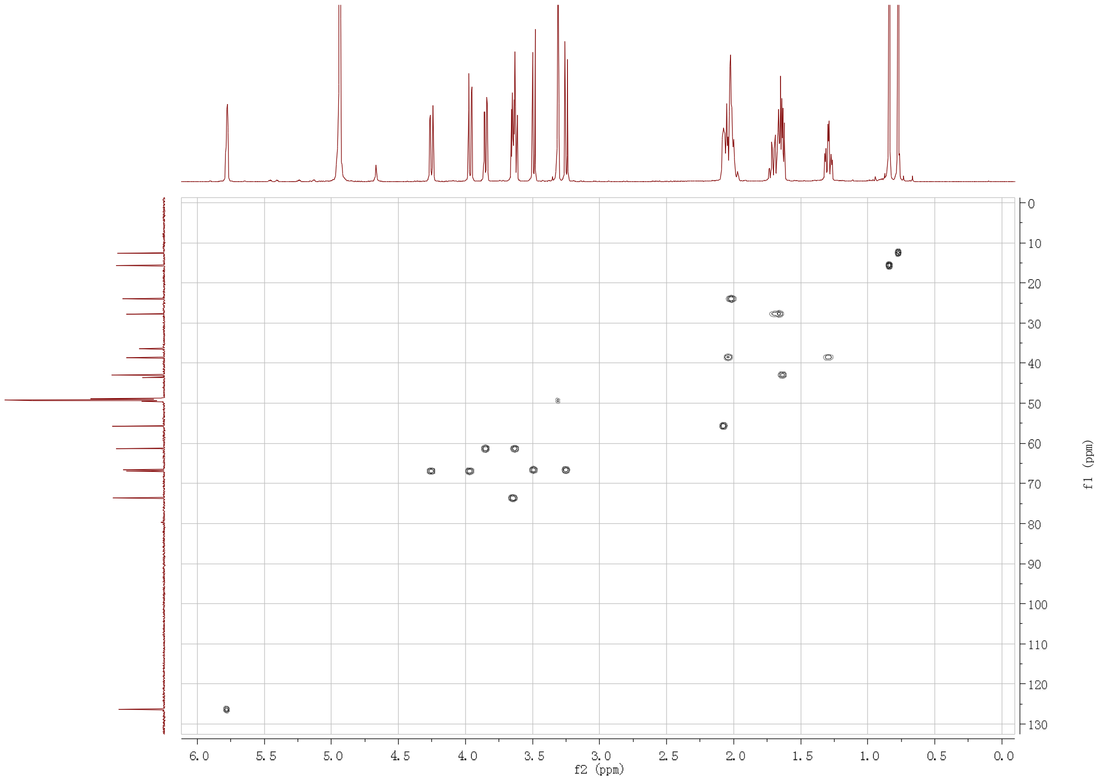

Figure S11. HMBC spectrum of phellinuin B (**2**)

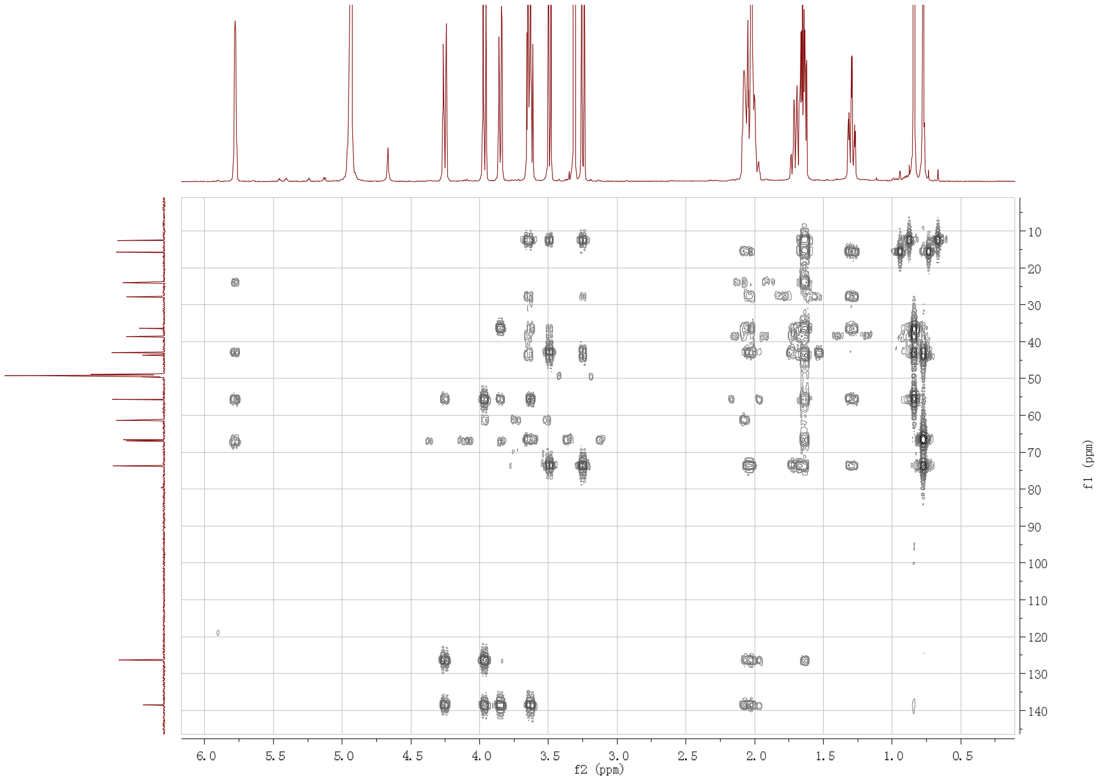

Figure S12. ^{1}H-^{1}H COSY spectrum of phellinuin B (**2**).

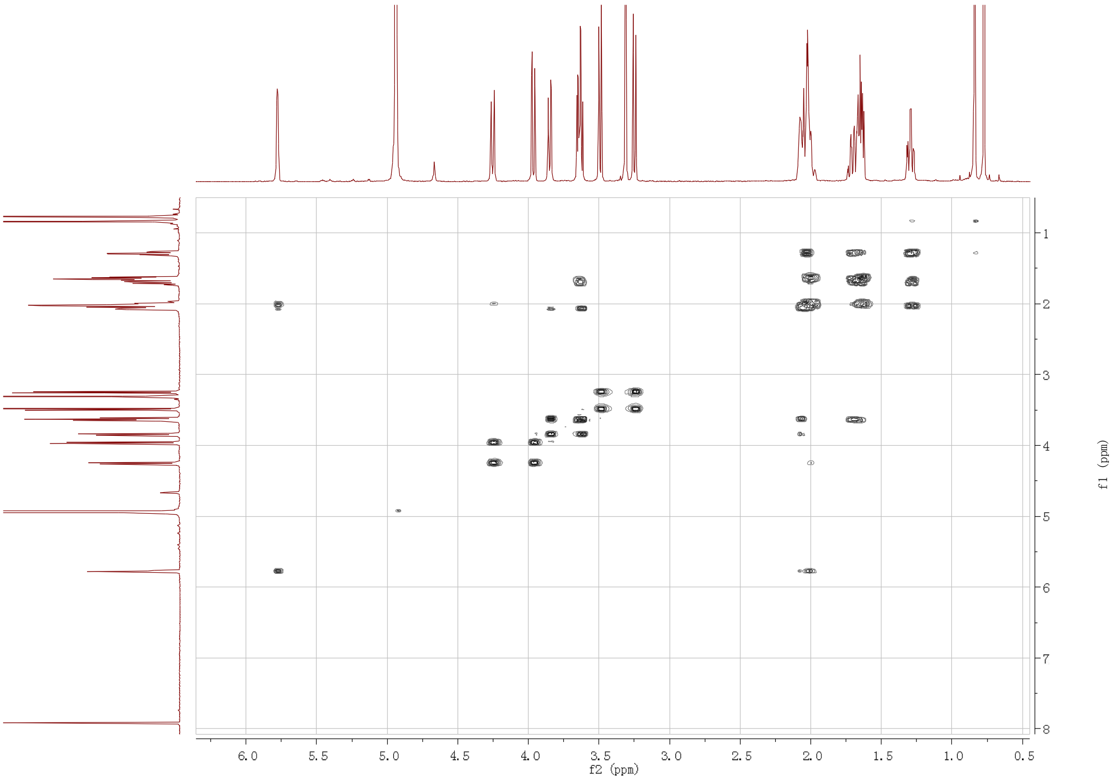

Figure S13. ROESY spectrum of phellinuin B (**2**).

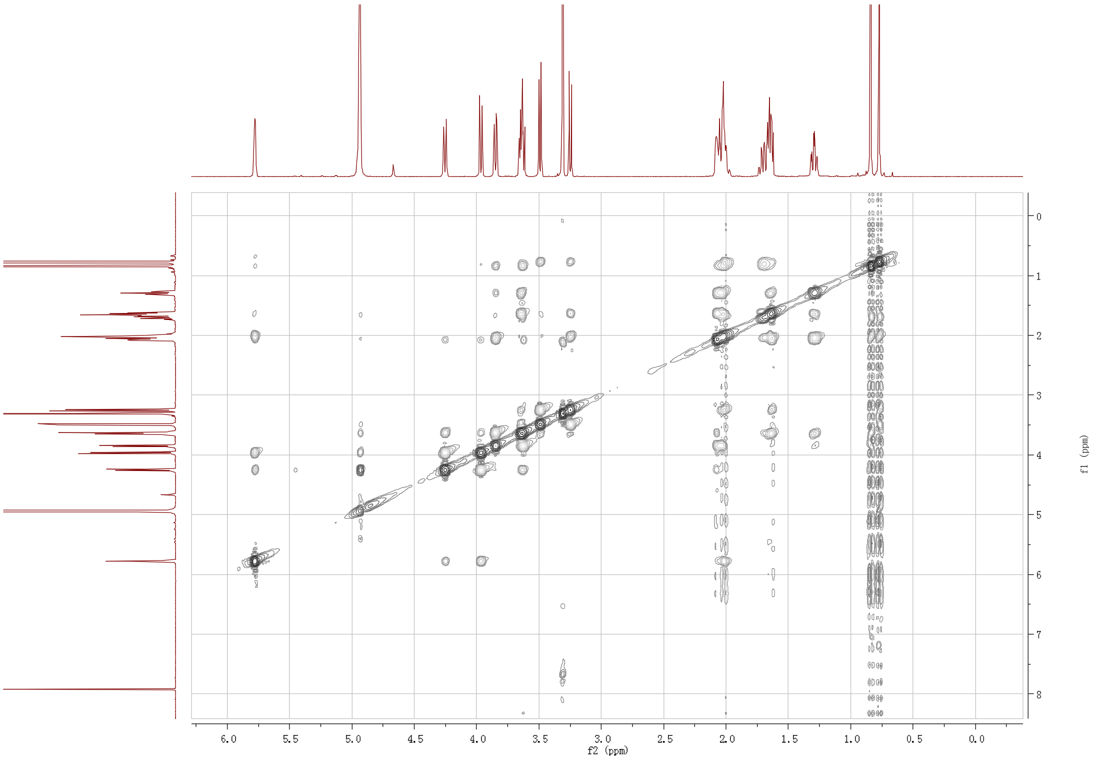

Figure S14. HREIMS of phellinuin B (**2**).

Elemental Composition Report **Page 1**

Single Mass Analysis
Tolerance = 10.0 PPM / DBE: min = -10.0, max = 120.0
Selected filters: None

Monoisotopic Mass, Odd and Even Electron Ions
14 formula(e) evaluated with 1 results within limits (up to 51 closest results for each mass)
Elements Used:
C: 0-200 H: 0-400 O: 3-5

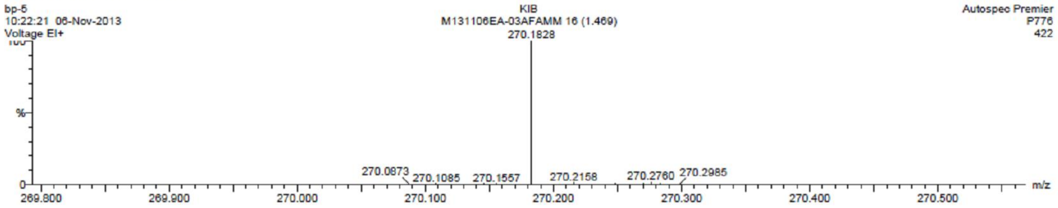

Minimum:				-10.0		
Maximum:		200.0	10.0	120.0		
Mass	Calc. Mass	mDa	PPM	DBE	i-FIT	Formula
270.1828	270.1831	-0.3	-1.1	3.0	5546215.0	C15 H26 O4

Figure S15. ^{1}H NMR (600 MHz, methanol-d_4) spectrum of phellinuin C (**3**).

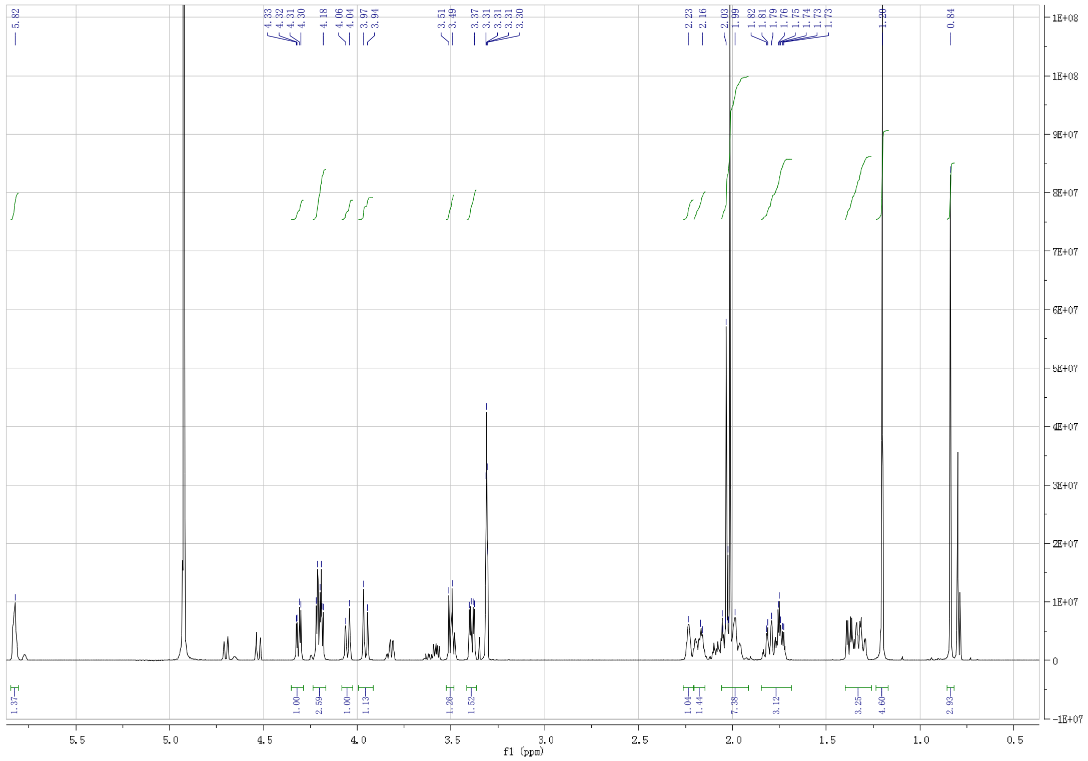

Figure S16. ^{13}C NMR and DEPT (150 MHz, methanol-d_4) spectrum of phellinuin C (**3**).

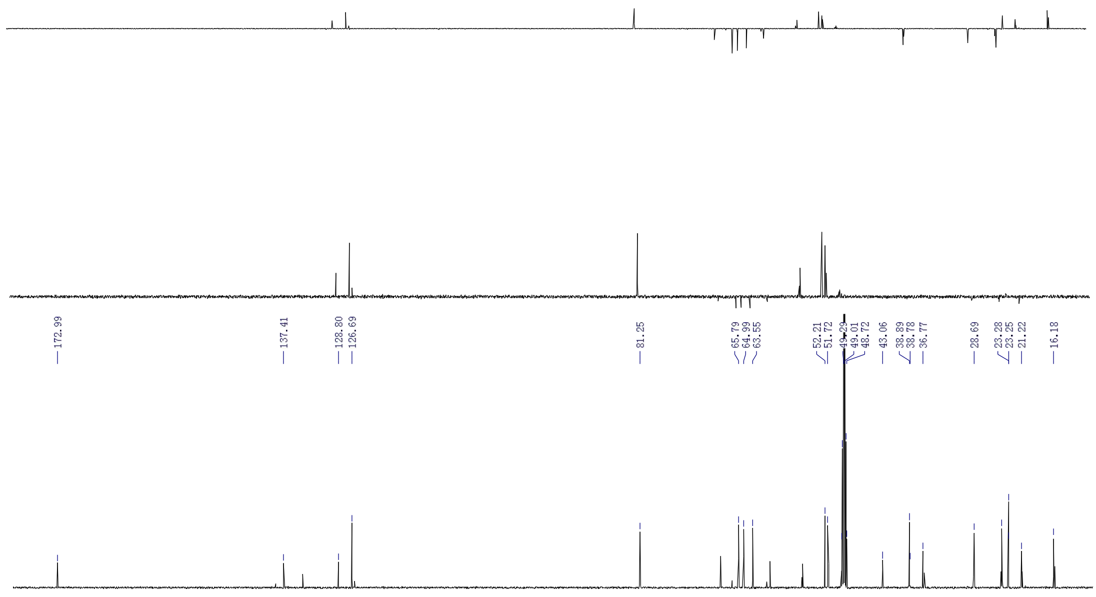

Figure S17. HSQC spectrum of phellinuin C (**3**).

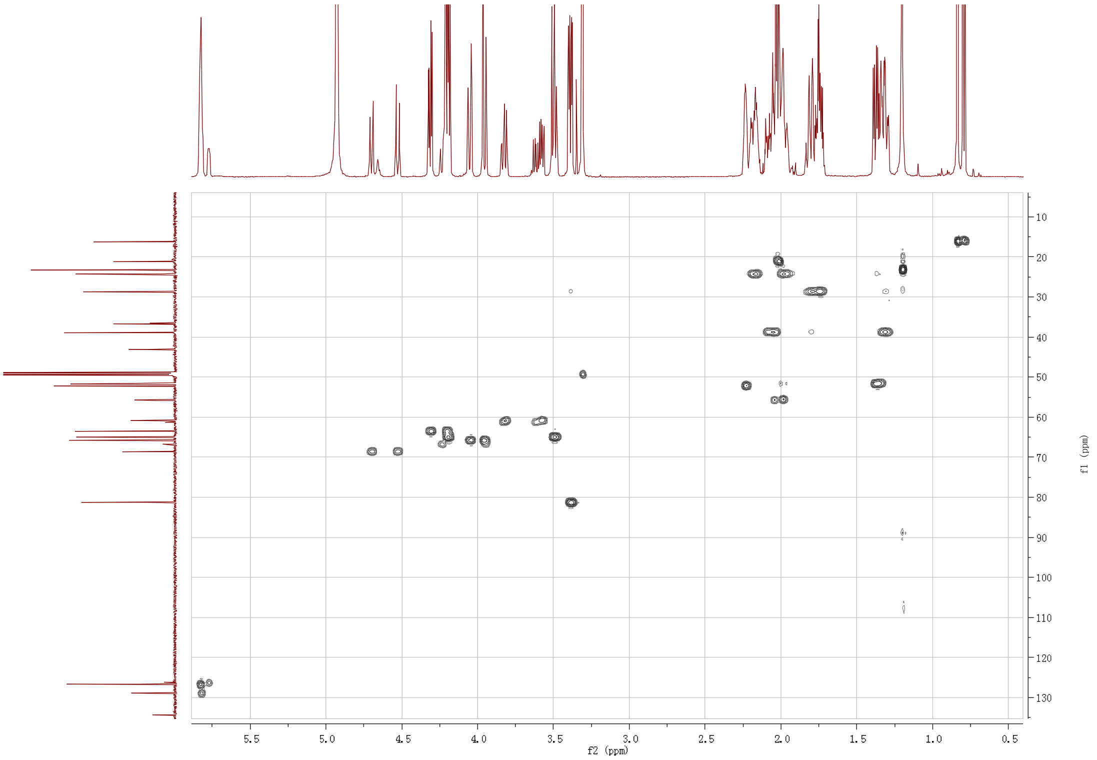

Figure S18. HMBC spectrum of phellinuin C (**3**).

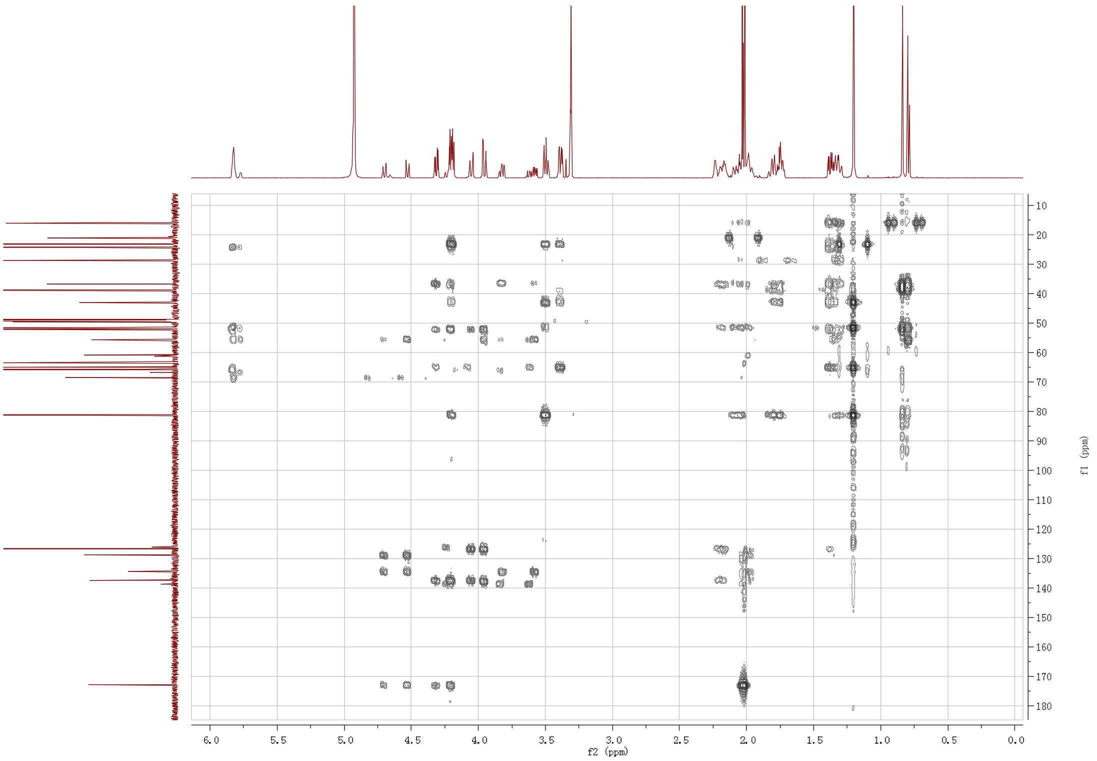

Figure S19. ^{1}H-^{1}H COSY spectrum of phellinuin C (**3**).

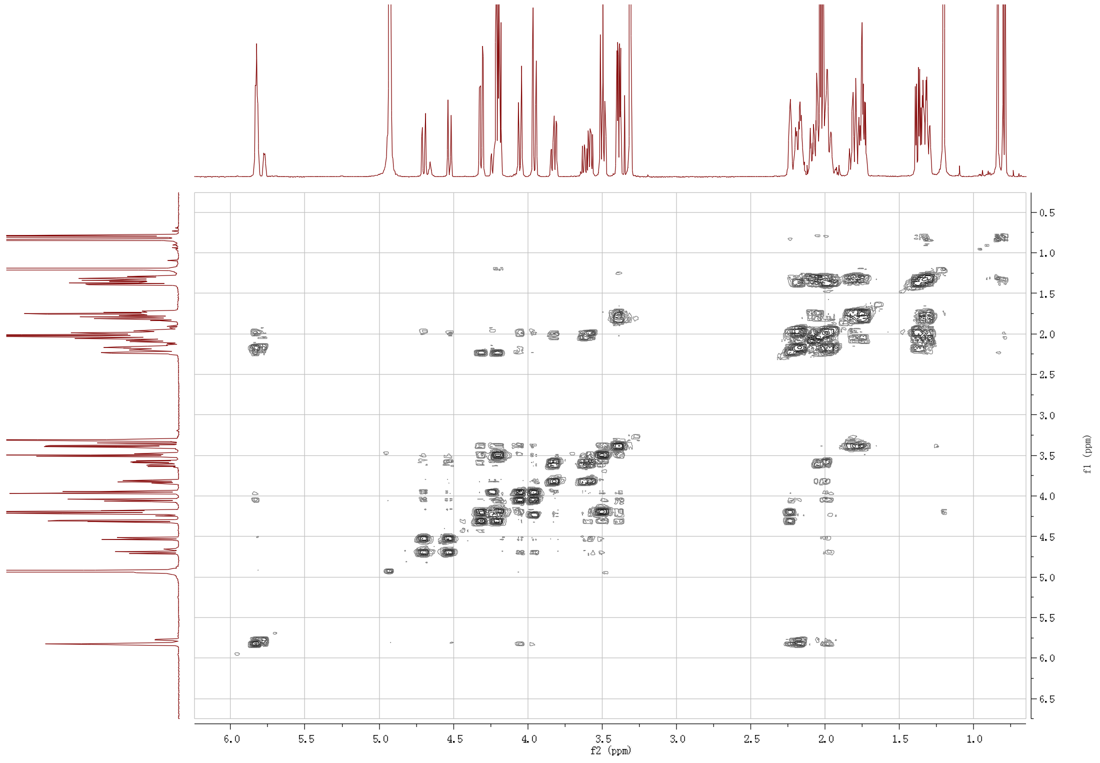

Figure S20. ROESY spectrum of phellinuin C (**3**).

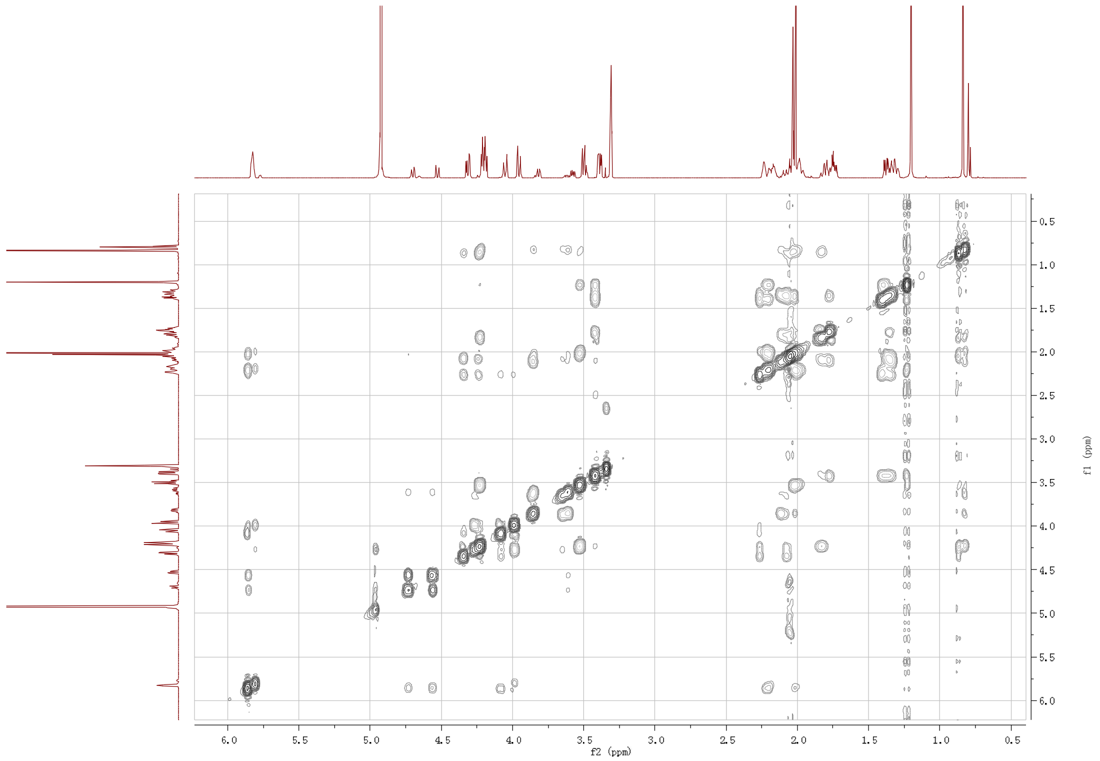

Figure S21. HREIMS of phellinuin C (**3**).

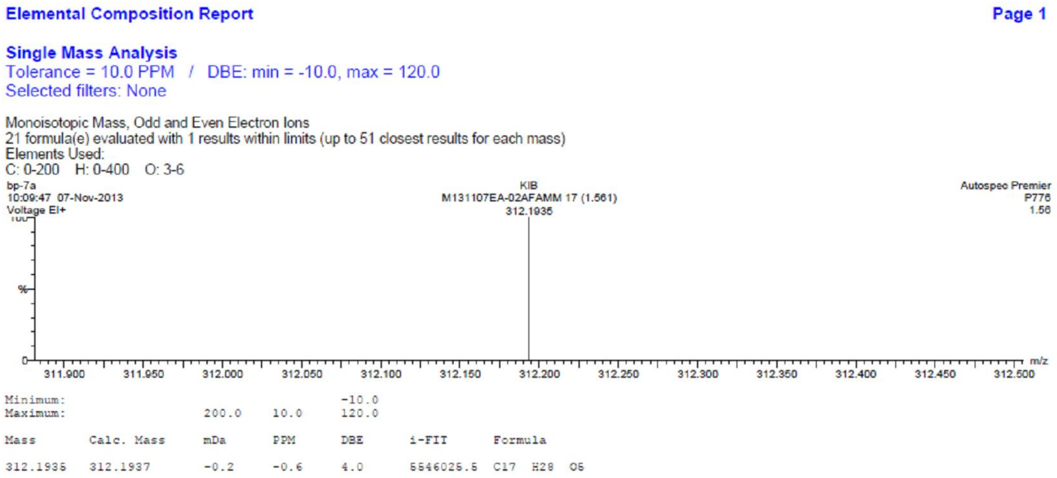

Figure S22. ^{1}H NMR (600 MHz, methanol-d_4) spectrum of phellinuin D (**4**).

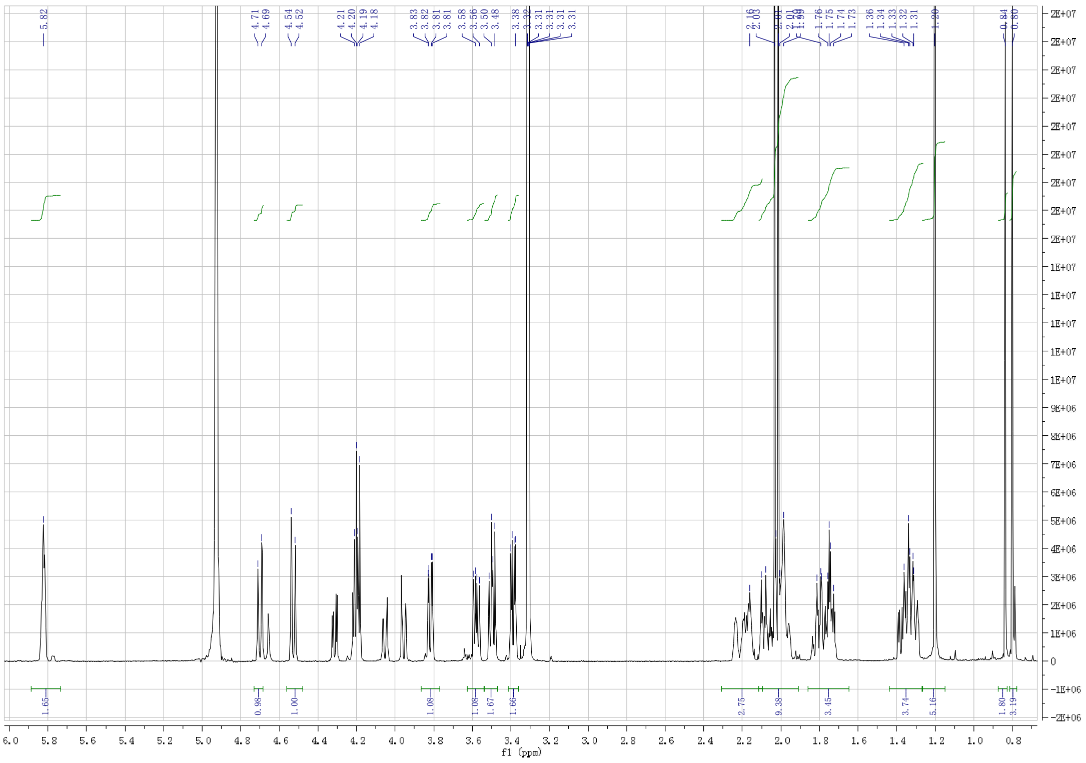

Figure S23. ^{13}C NMR and DEPT (150 MHz, methanol-d_4) spectrum of phellinuin D (**4**).

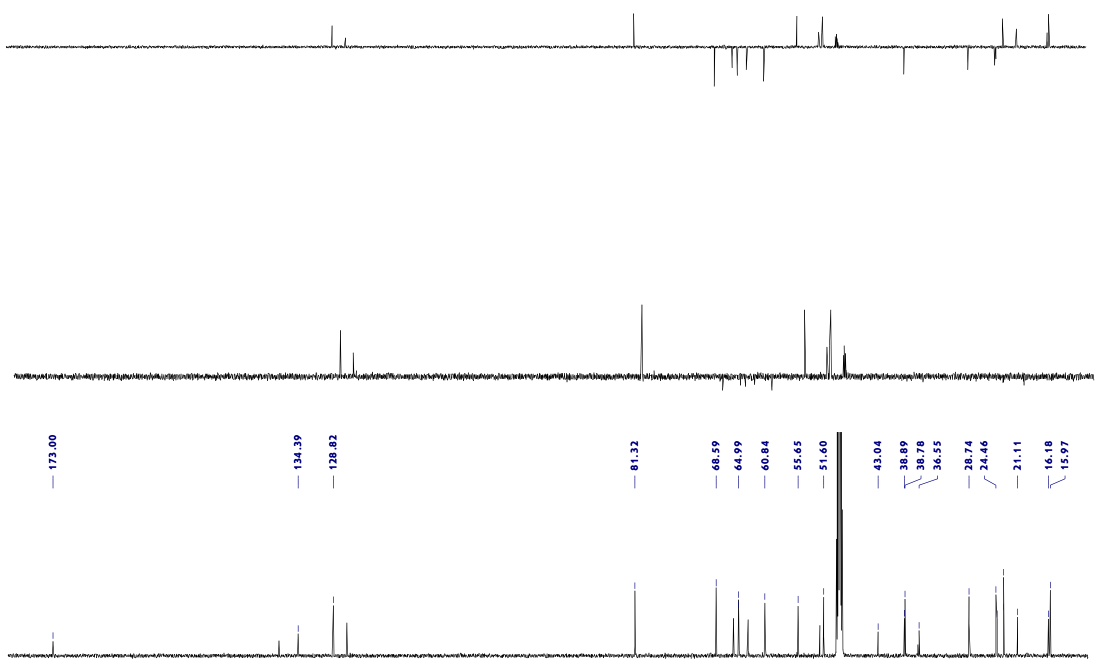

Figure S24. HSQC spectrum of phellinuin D (**4**).

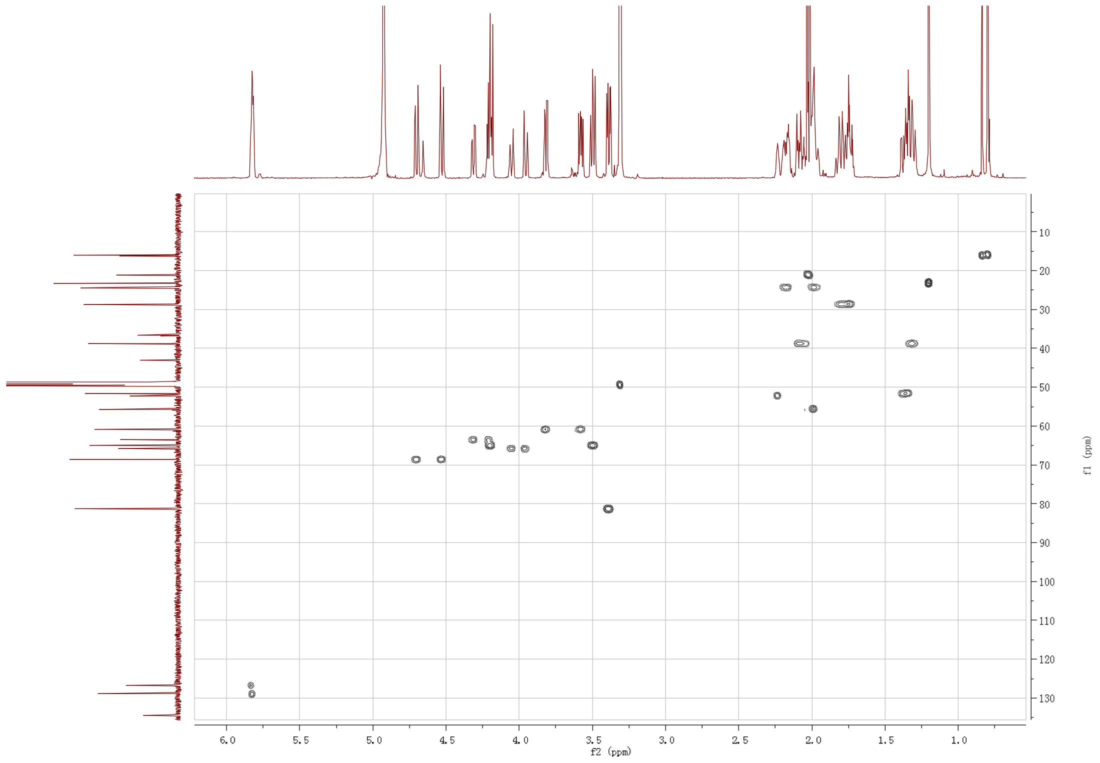

Figure S25. HMBC spectrum of phellinuin D (**4**).

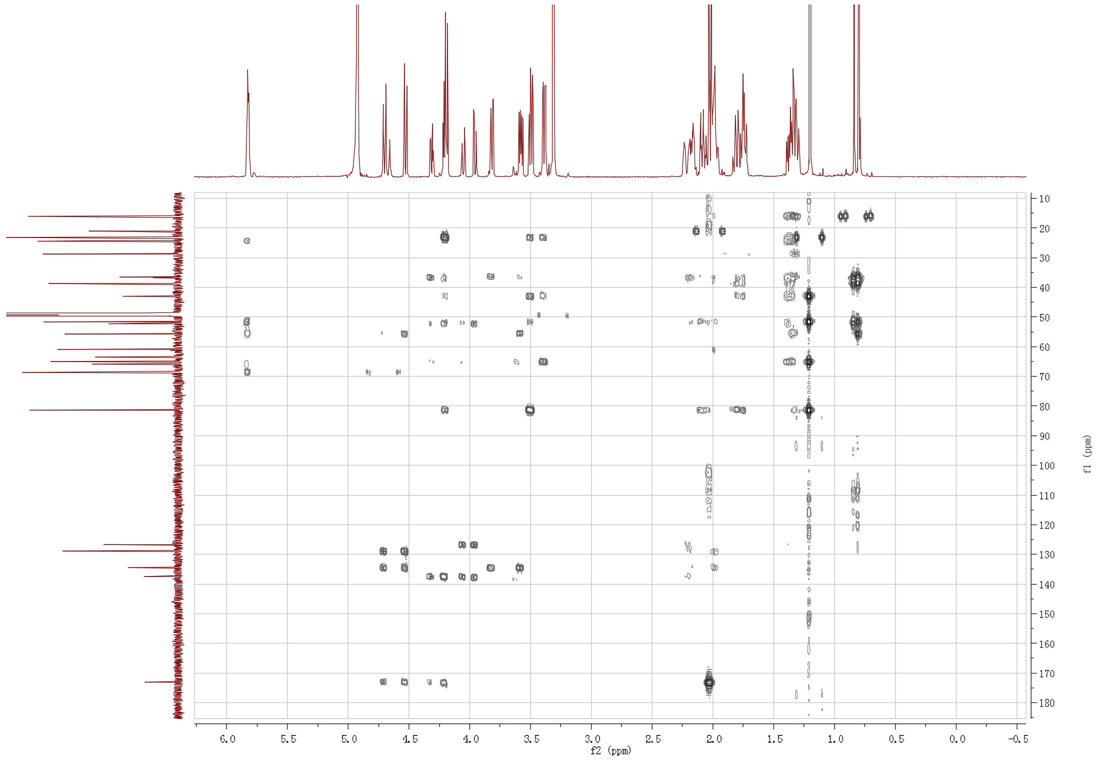

Figure S26. ^{1}H-^{1}H COSY spectrum of phellinuin D (**4**).

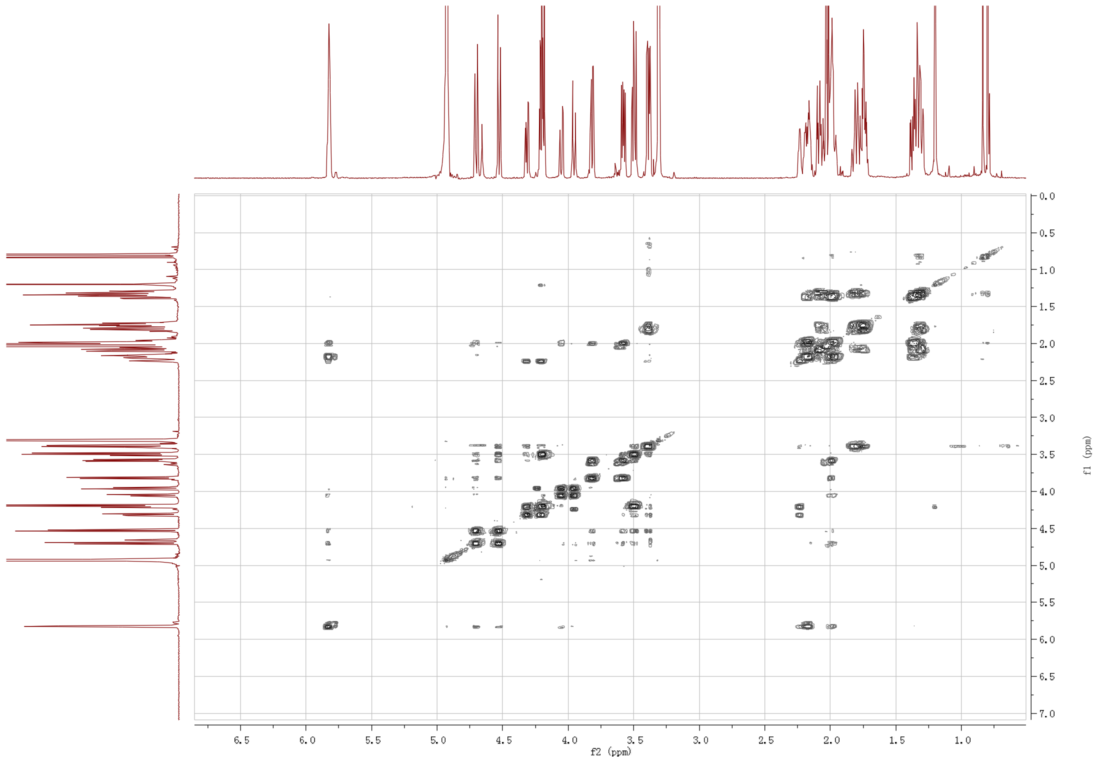

Figure S27. ROESY spectrum of phellinuin D (**4**).

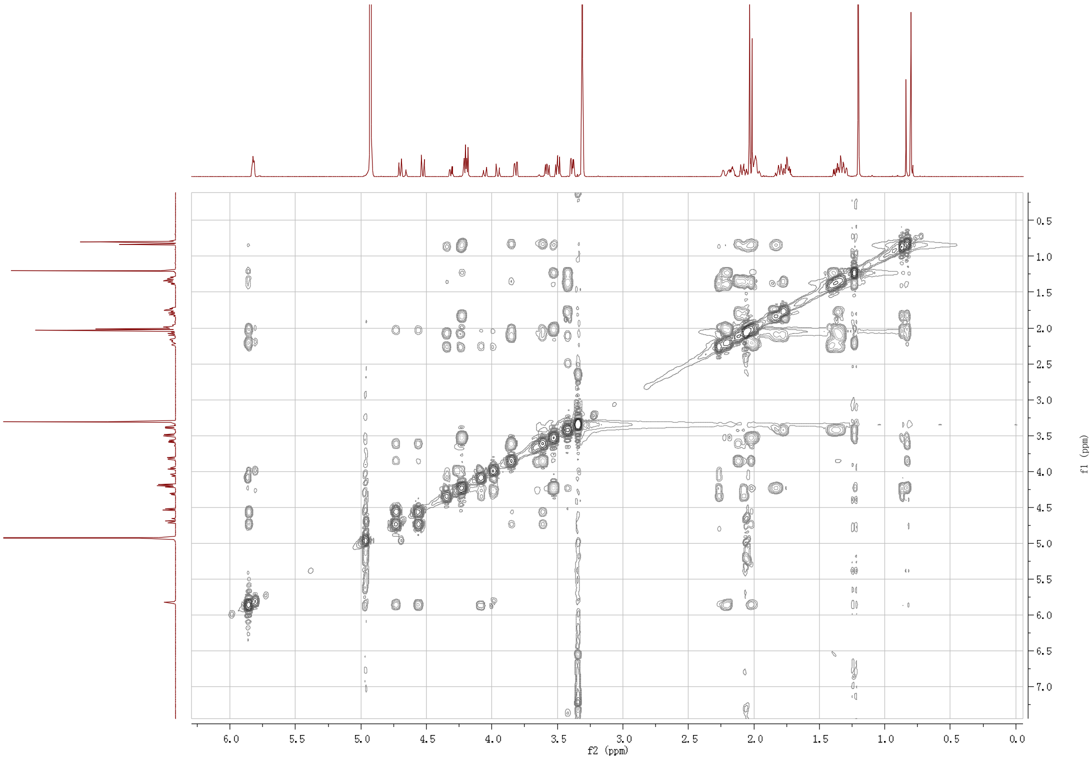

Figure S28. HREIMS of phellinuin D (**4**).

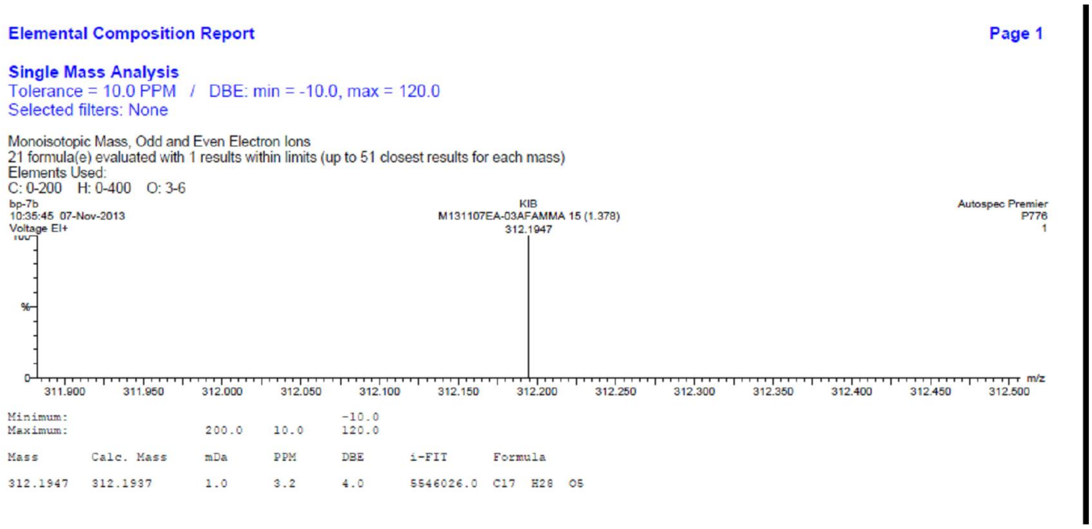

Elemental Composition Report **Page 1**

Single Mass Analysis
Tolerance = 10.0 PPM / DBE: min = -10.0, max = 120.0
Selected filters: None

Monoisotopic Mass, Odd and Even Electron Ions
21 formula(e) evaluated with 1 results within limits (up to 51 closest results for each mass)
Elements Used:
C: 0-200 H: 0-400 O: 3-6

bp-7b
10:35:45 07-Nov-2013
Voltage EI+

KIB
M131107EA-03AFAMMA 15 (1.378)
312.1947

Autospec Premier
P776
1

Minimum:				-10.0		
Maximum:		200.0	10.0	120.0		
Mass	Calc. Mass	mDa	PPM	DBE	i-FIT	Formula
312.1947	312.1937	1.0	3.2	4.0	5546026.0	C17 H28 O5

Figure S29. ^{1}H NMR (600 MHz, methanol-d_4) spectrum of phellinuin E (**5**).

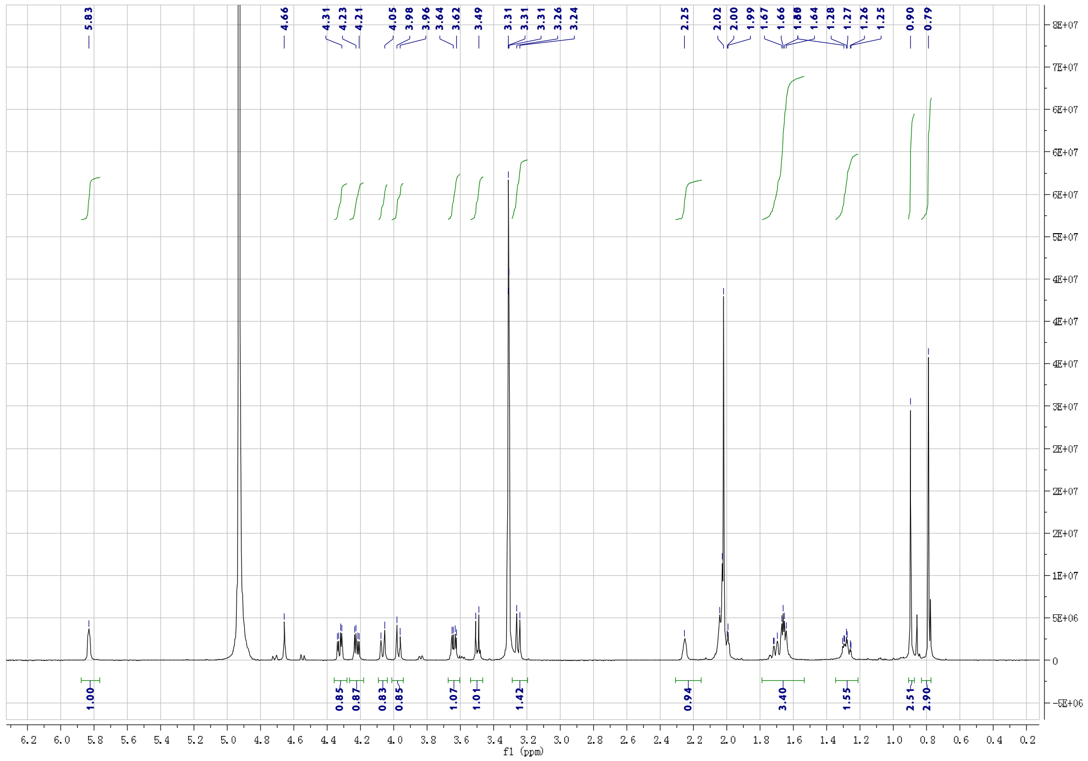

Figure S30. ^{13}C NMR and DEPT (150 MHz, methanol-d_4) spectrum of phellinuin E (**5**).

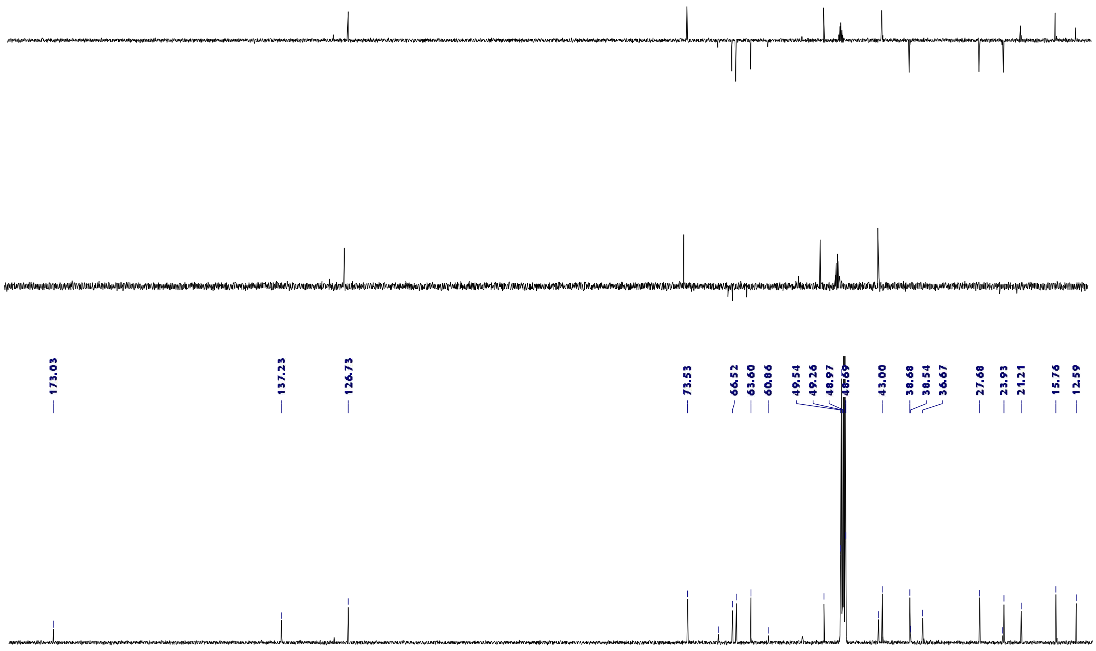

Figure S31. HSQC spectrum of phellinuin E (**5**).

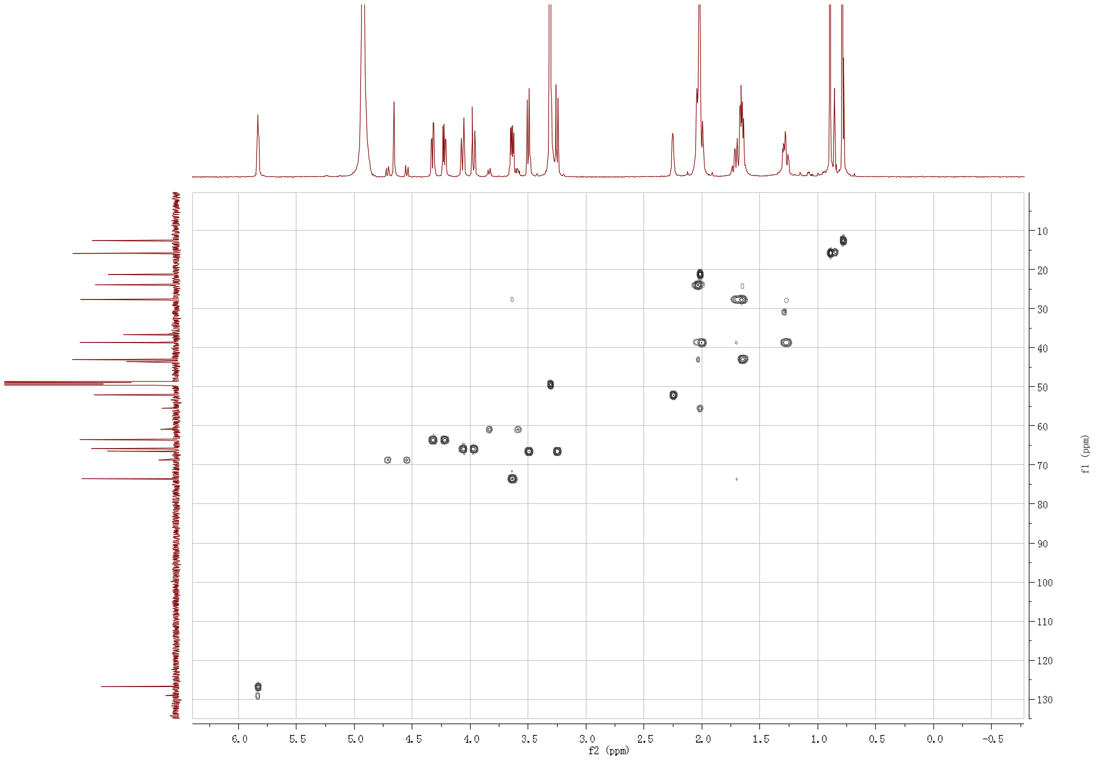

Figure S32. HMBC spectrum of phellinuin E (**5**).

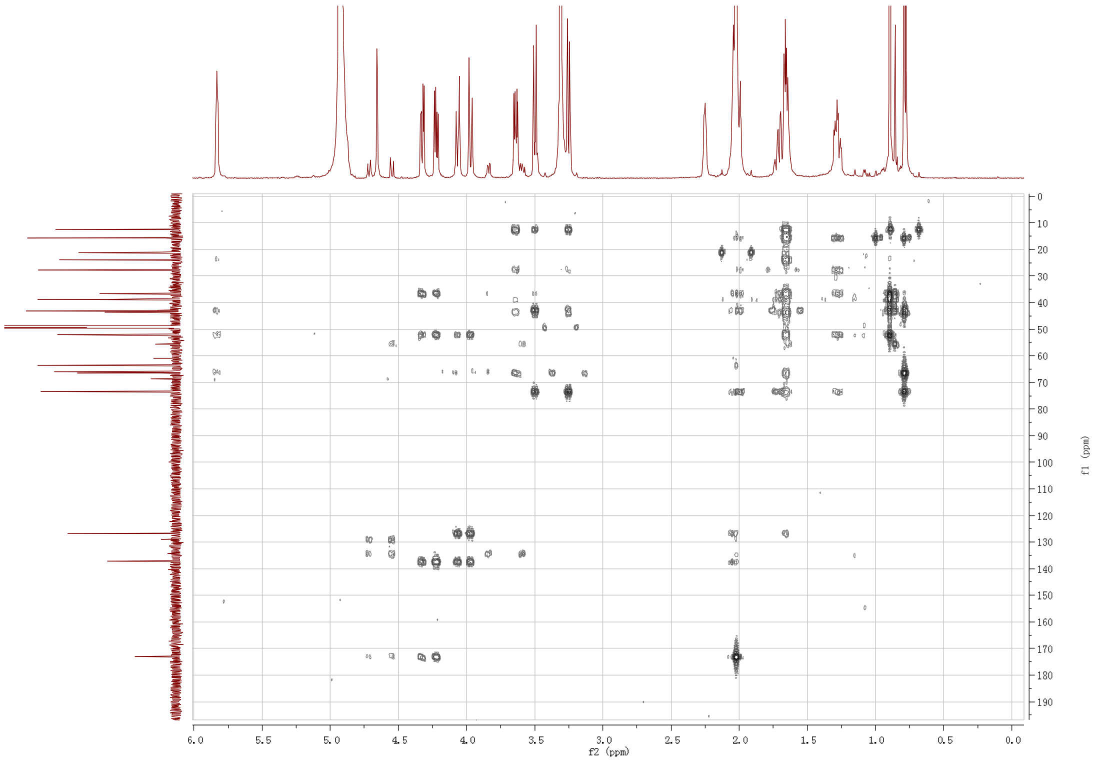

Figure S33. ^{1}H-^{1}H COSY spectrum of phellinuin E (**5**).

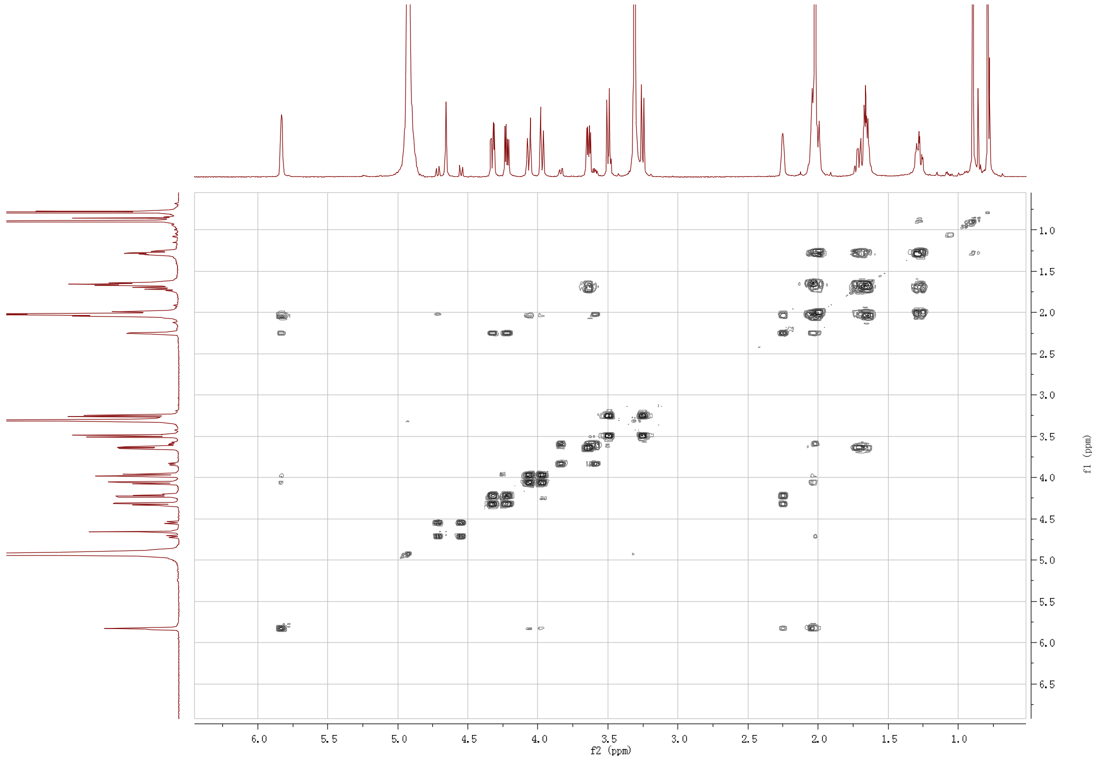

Figure S34. ROESY spectrum of phellinuin E (**5**).

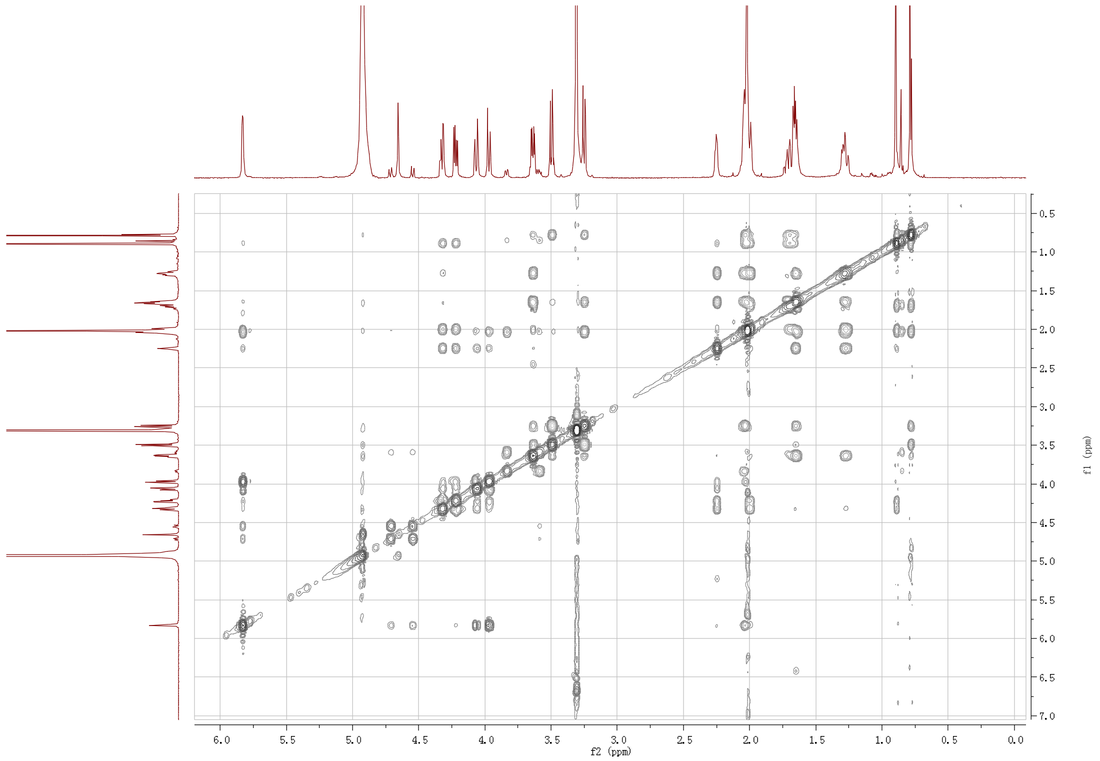

Figure S35. HREIMS of phellinuin E (**5**).

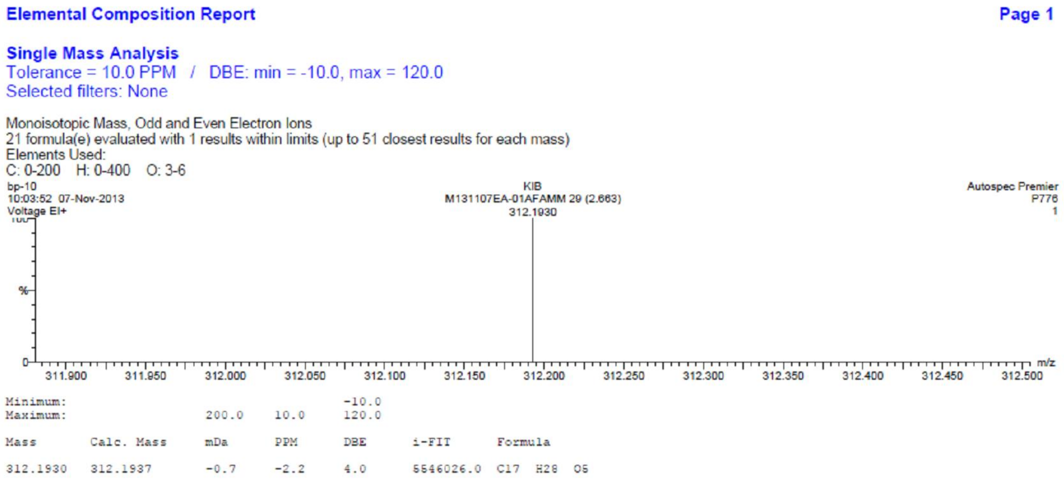

Figure S36. ^{1}H NMR (500 MHz, methanol-d_4) spectrum of phellinuin F (**6**).

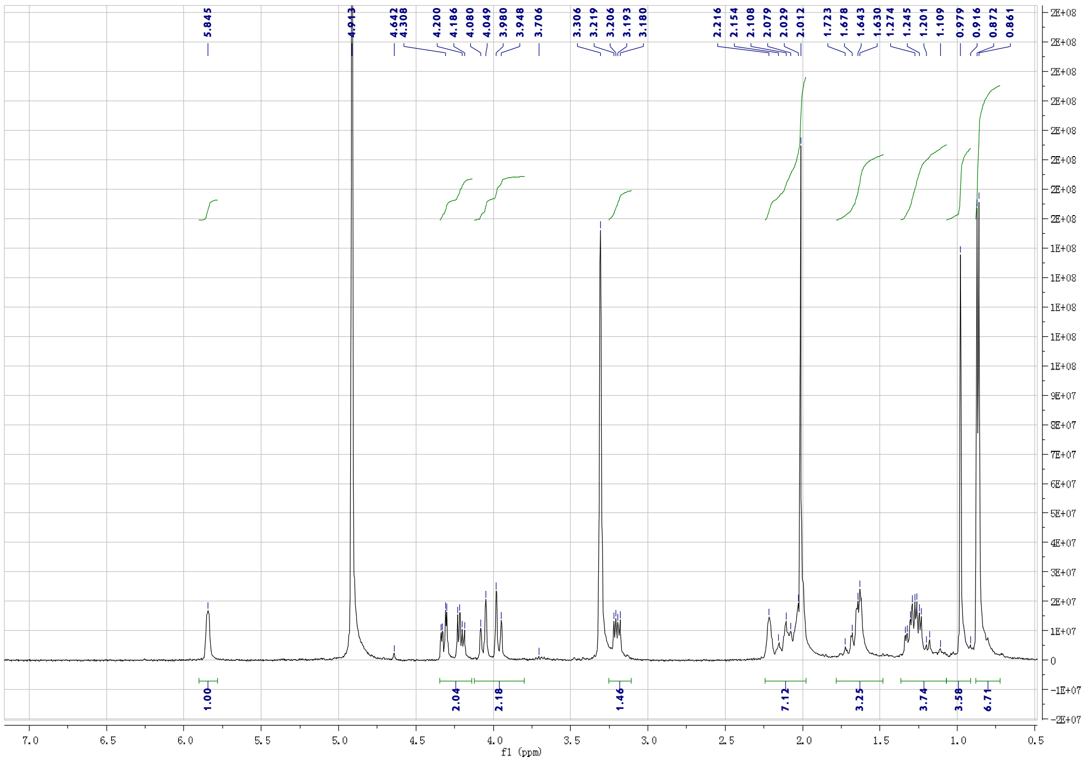

Figure S37. ^{13}C NMR and DEPT (125 MHz, methanol-d_4) spectrum of phellinuin F (**6**).

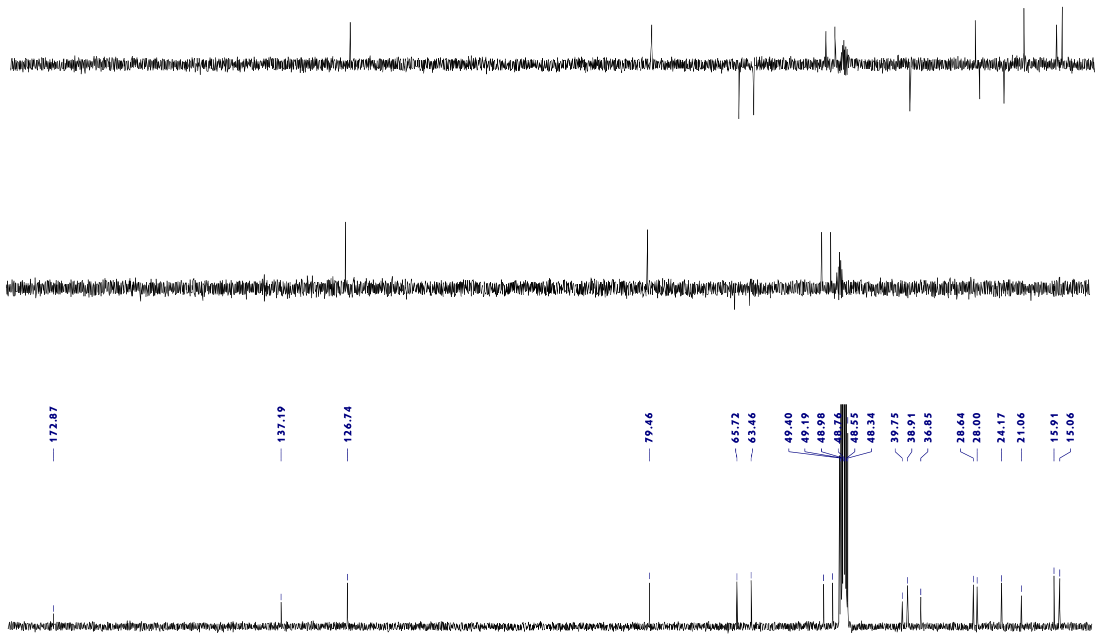

Figure S38. HSQC spectrum of phellinuin F (**6**).

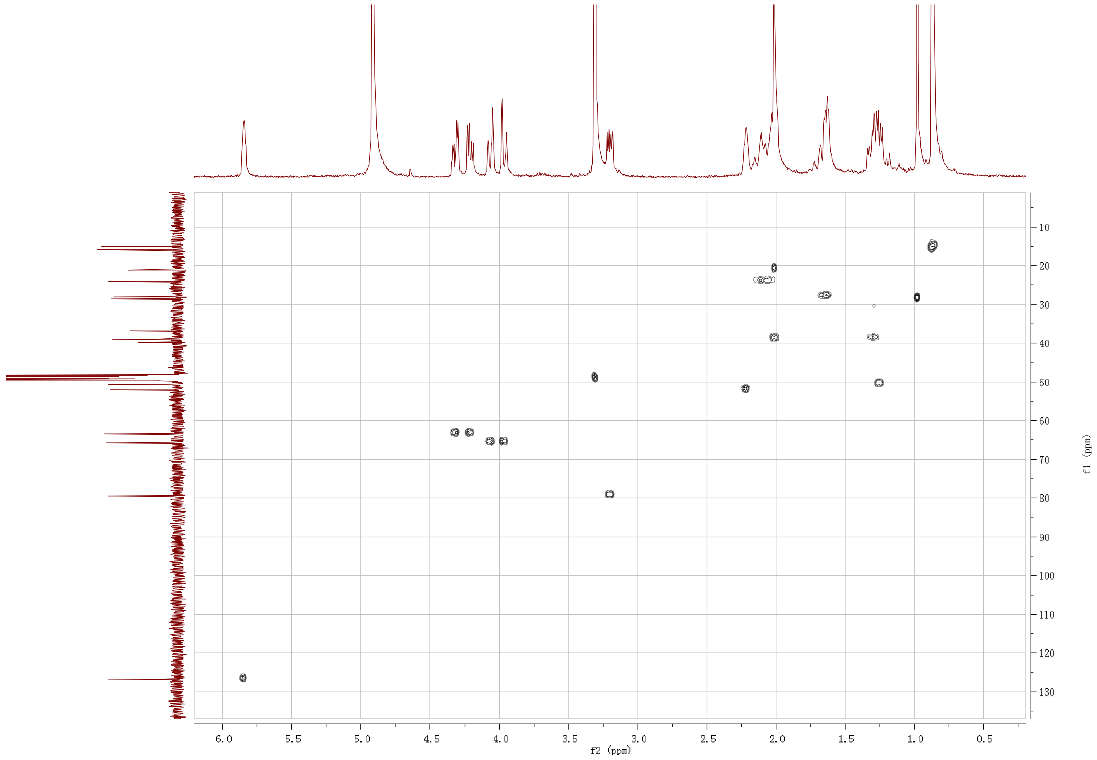

Figure S39. HMBC spectrum of phellinuin F (**6**).

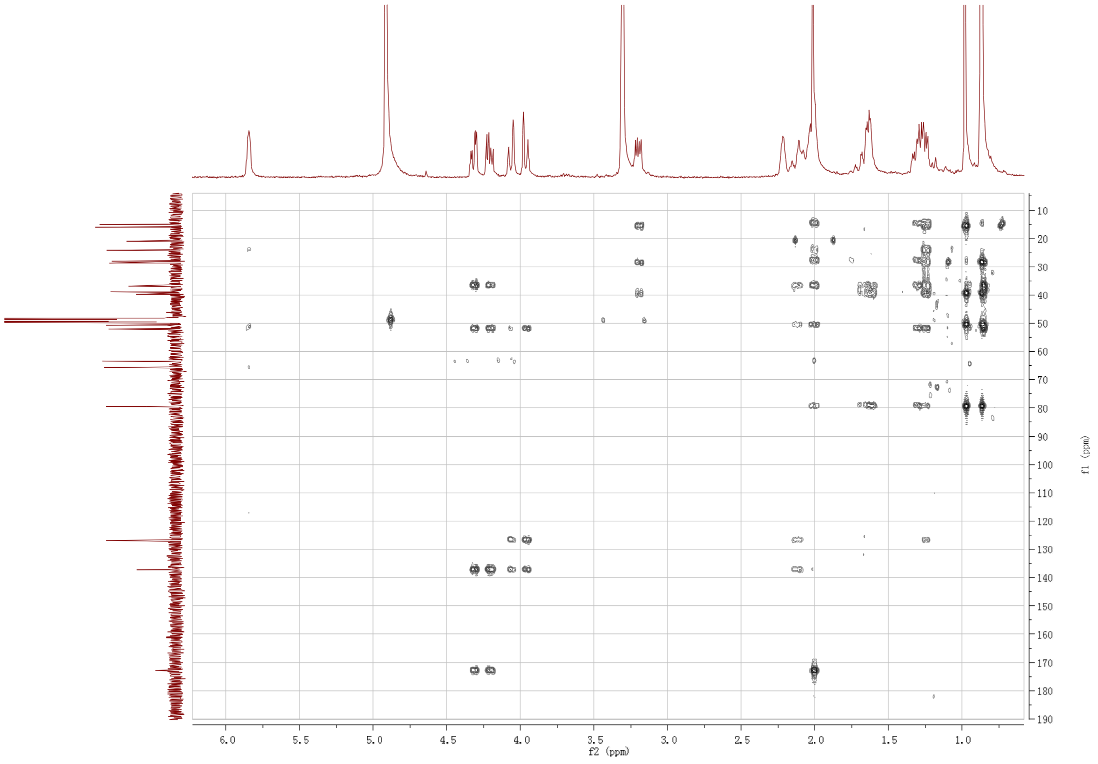

Figure S40. ^{1}H-^{1}H COSY spectrum of phellinuin F (**6**).

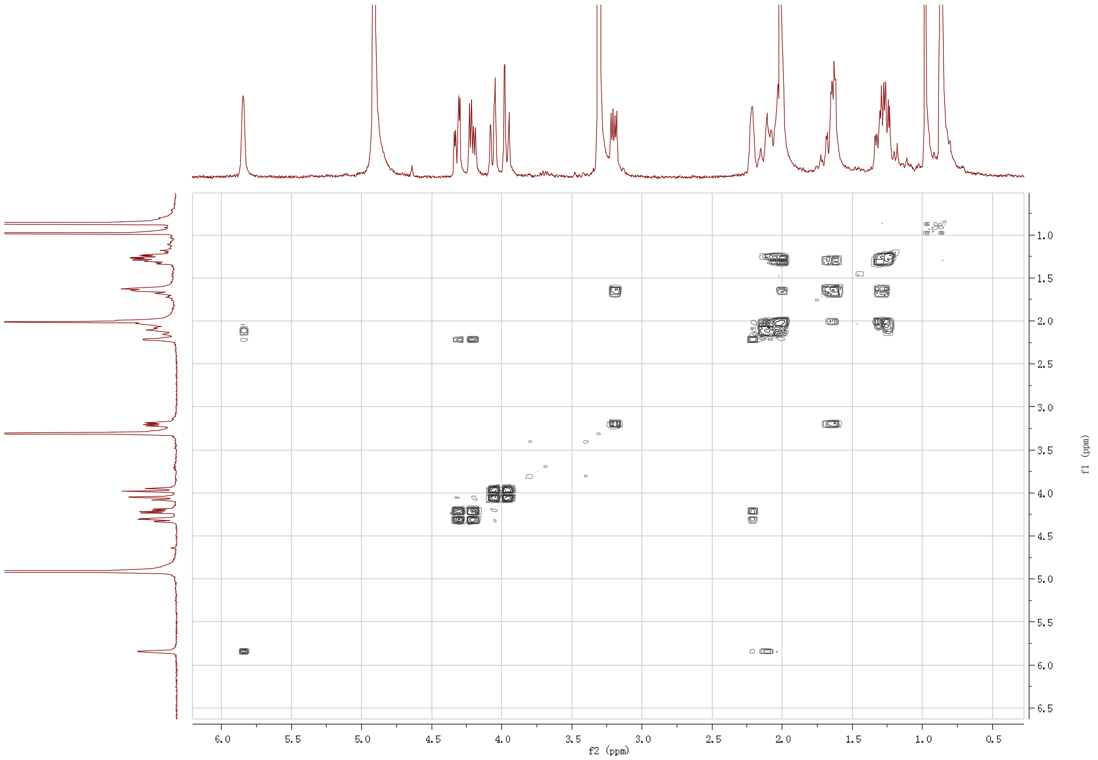

Figure S41. ROESY spectrum of phellinuin F (**6**).

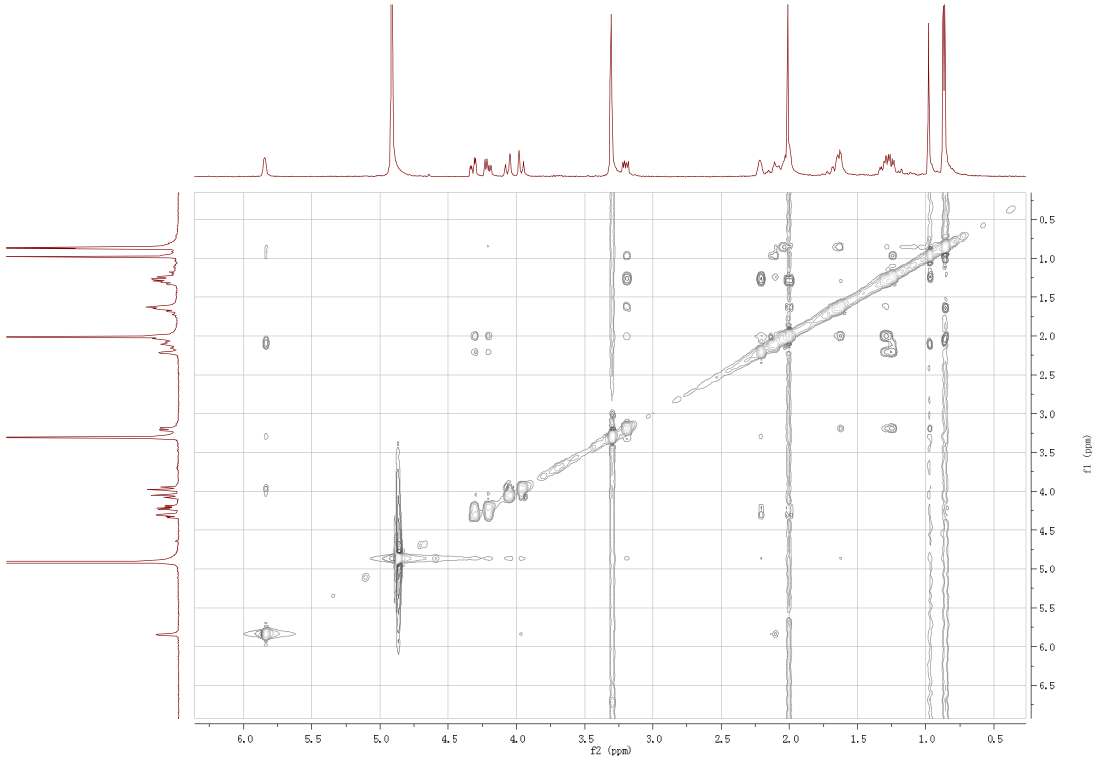

Figure S42. HREIMS of phellinuin F (**6**).

Elemental Composition Report **Page 1**

Single Mass Analysis
Tolerance = 10.0 PPM / DBE: min = -10.0, max = 120.0
Selected filters: None

Monoisotopic Mass, Odd and Even Electron Ions
19 formula(e) evaluated with 1 results within limits (up to 51 closest results for each mass)
Elements Used:
C: 0-200 H: 0-400 O: 3-6

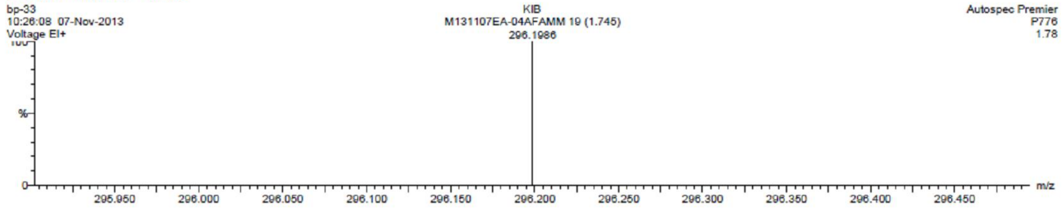

	Calc. Mass	mDa	PPM	DBE	i-FIT	Formula
Minimum:				-10.0		
Maximum:		200.0	10.0	120.0		
**Mass**						
296.1986	296.1988	-0.2	-0.7	4.0	5546025.5	C17 H28 O4

Figure S43. ^{1}H NMR (600 MHz, methanol-d_4) spectrum of phellinuin G (**7**).

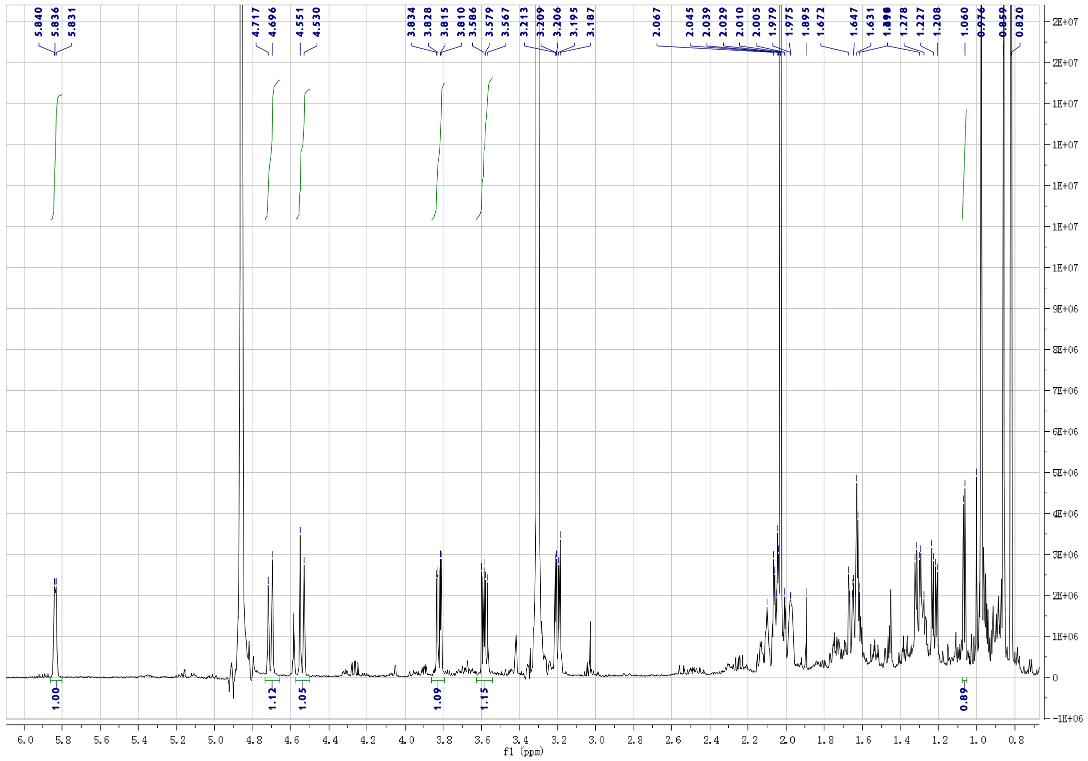

Figure S44. ^{13}C NMR and DEPT (150 MHz, methanol-d_4) spectrum of phellinuin G (**7**).

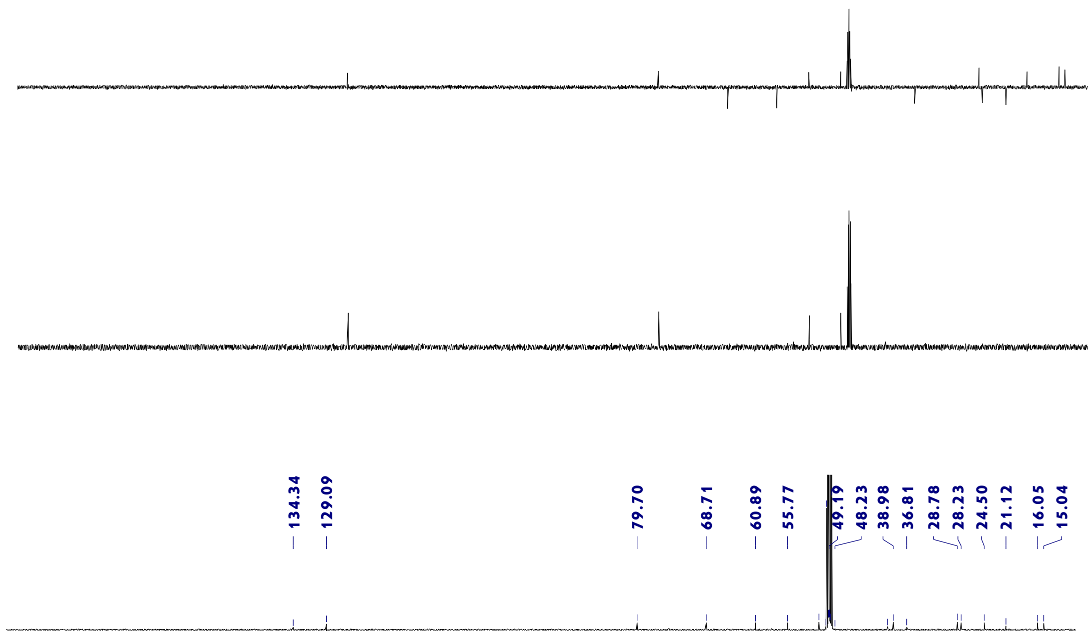

Figure S45. HSQC spectrum of phellinuin G (**7**).

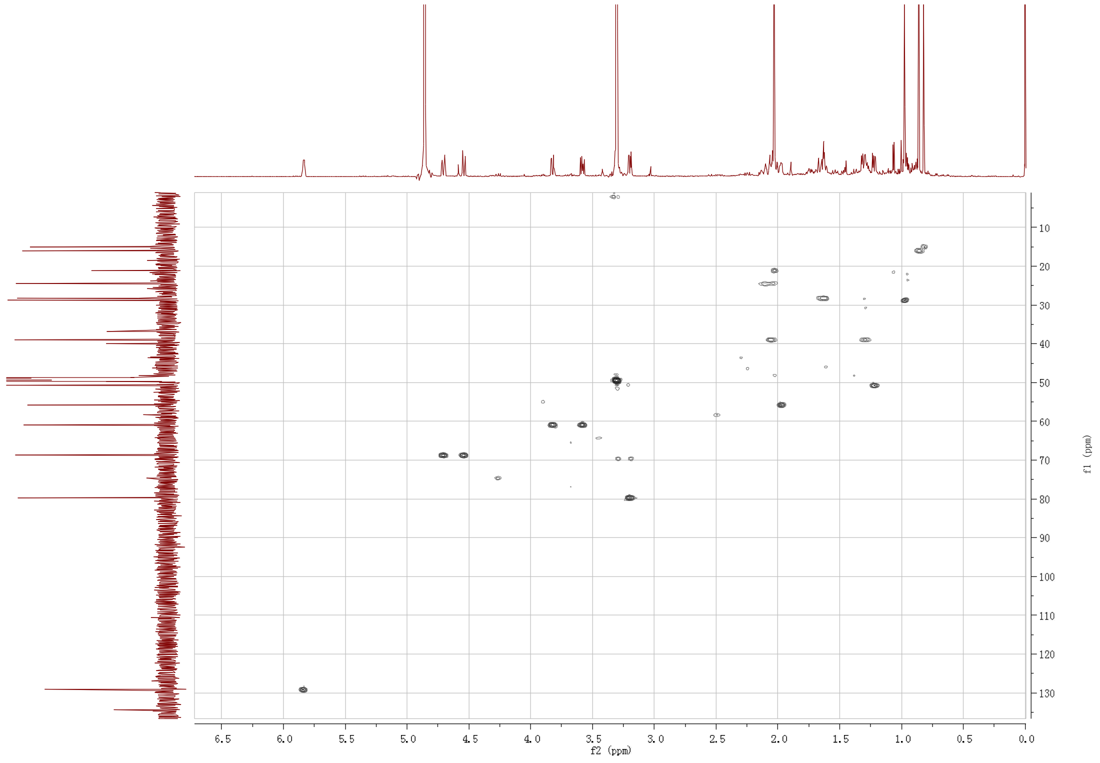

Figure S46. HMBC spectrum of phellinuin G (**7**).

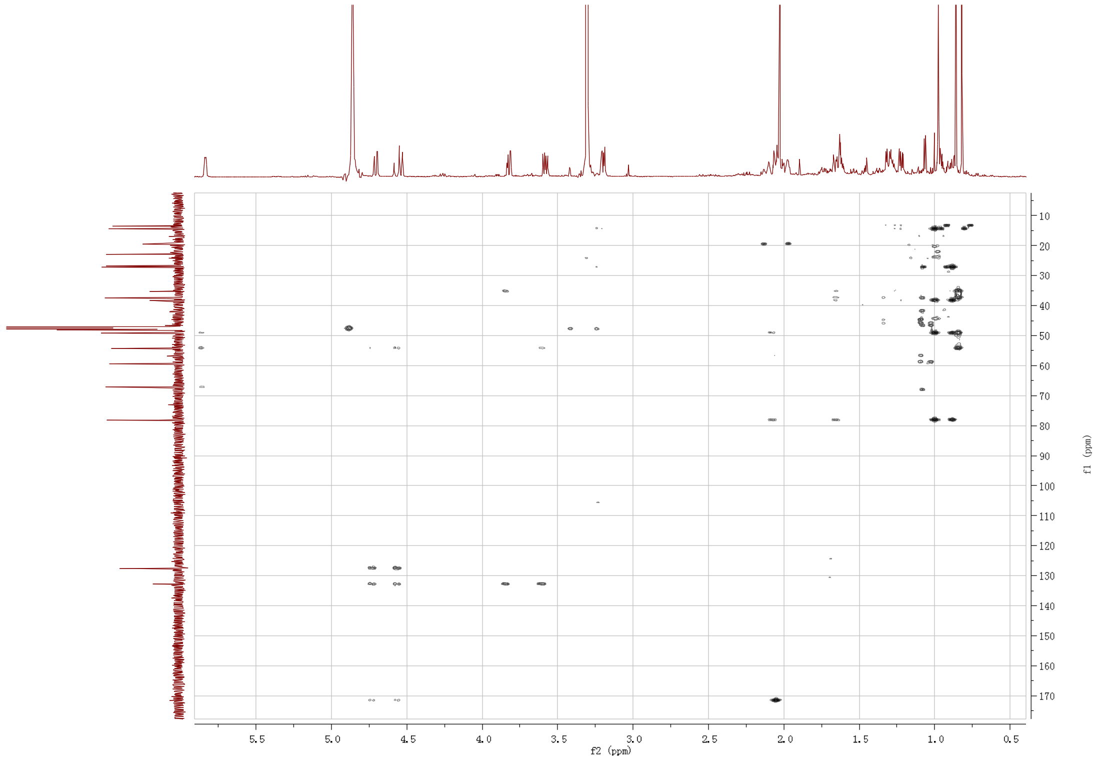

Figure S47. ^{1}H-^{1}H COSY spectrum of phellinuin G (**7**).

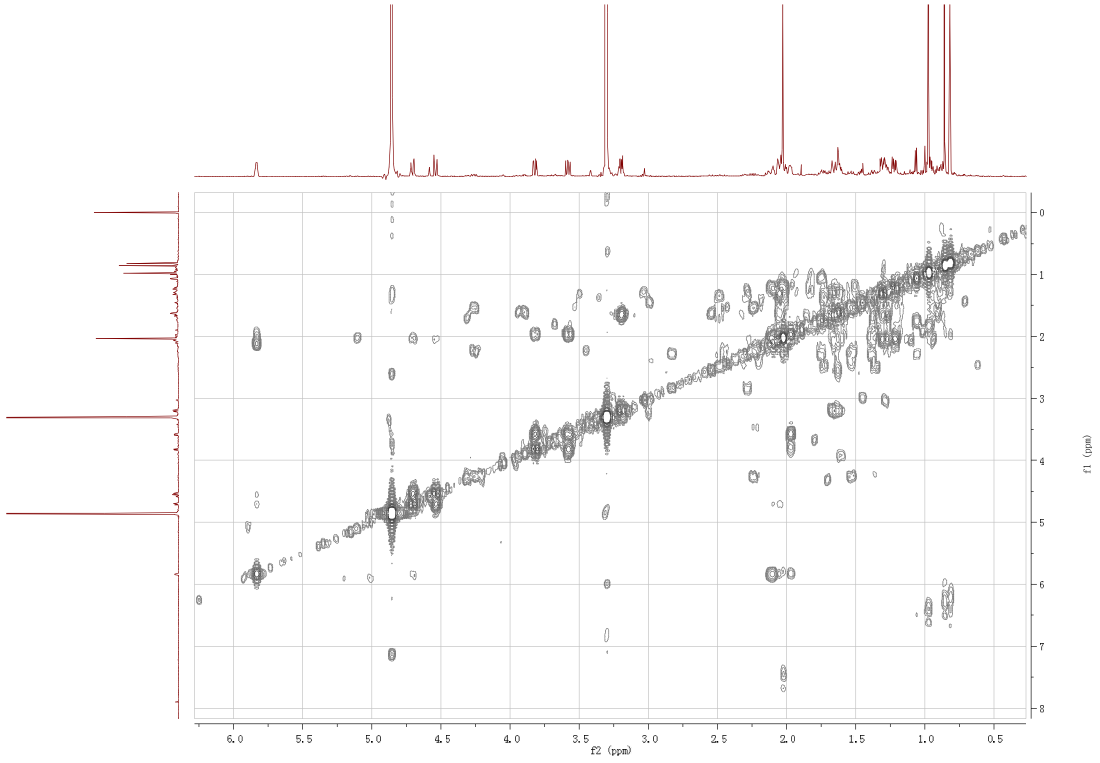

Figure S48. ROESY spectrum of phellinuin G (**7**).

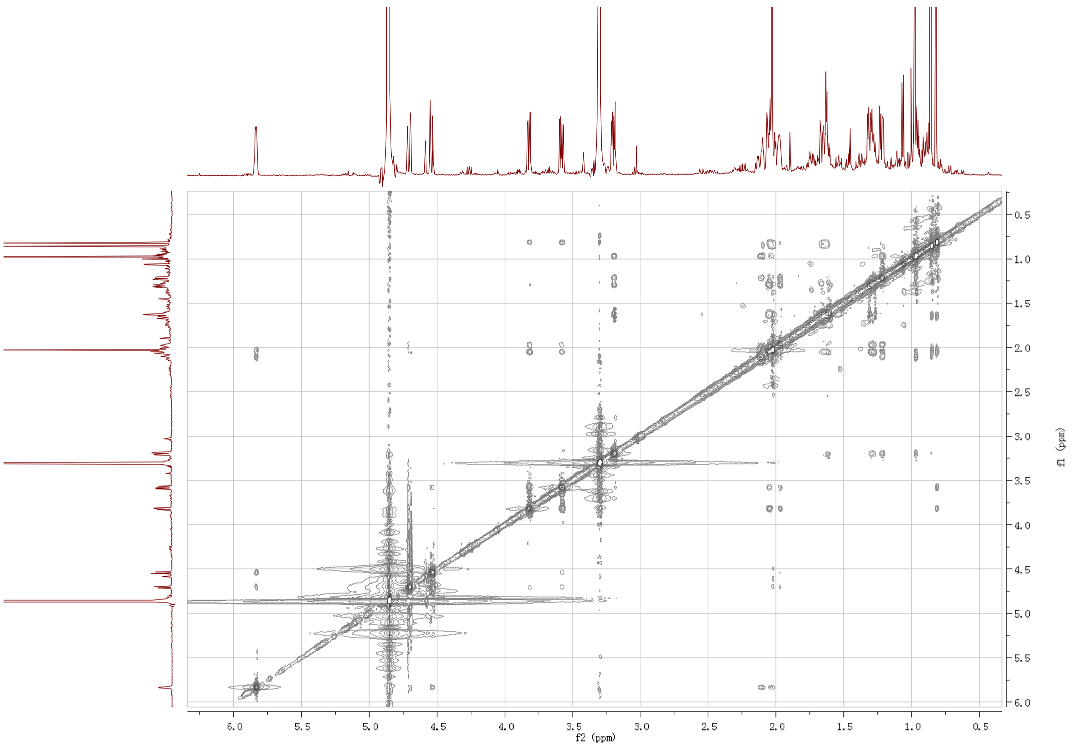

.

Figure S49. HREIMS of phellinuin G (**7**).

Elemental Composition Report **Page 1**

Single Mass Analysis
Tolerance = 10.0 PPM / DBE: min = -10.0, max = 120.0
Selected filters: None

Monoisotopic Mass, Odd and Even Electron Ions
19 formula(e) evaluated with 1 results within limits (up to 51 closest results for each mass)
Elements Used:
C: 0-200 H: 0-400 O: 3-6

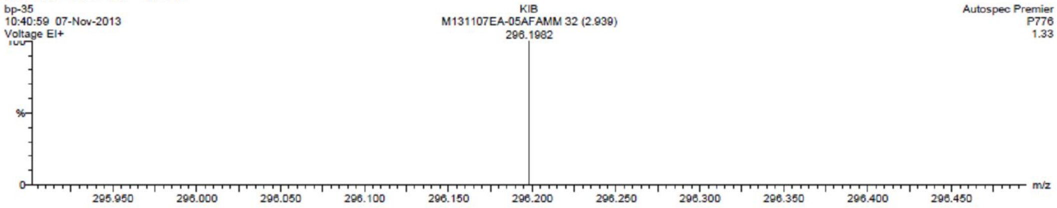

Minimum:				-10.0		
Maximum:		200.0	10.0	120.0		
Mass	Calc. Mass	mDa	PPM	DBE	i-FIT	Formula
296.1982	296.1988	-0.6	-2.0	4.0	5546025.5	C17 H28 O4
